# Exploiting coordination geometry to tune the dimensions and processability of metallosupramolecular polymers†

**DOI:** 10.1039/d1qo00644d

**Published:** 2021-06-04

**Authors:** Nils Bäumer, Kalathil K. Kartha, Stefan Buss, Jasnamol P. Palakkal, Cristian A. Strassert, Gustavo Fernández

**Affiliations:** Organisch Chemisches Institut, Universität Münster Corrensstraße 36 48149 Münster Germany fernandg@uni-muenster.de; Institut für Anorganische und Analytische Chemie, CiMIC, SoN, Westfälische Wilhelms-Universität Münster Corrensstraße 28/30 48149 Münster Germany; CeNTech, Westfälische Wilhelms-Universität Münster Heisenbergstraße 11 48149 Münster Germany; Institute of Materials Science, Technische Universität Darmstadt 64287 Darmstadt Germany

## Abstract

Achieving precise control over the morphology, dimensions and processability of functional materials is a key but challenging requirement for the fabrication of smart devices. To address this issue, we herein compare the self-assembly behavior of two new Pt(ii) complexes that differ in the molecular and coordination geometry through implementation of either a monodentate (pyridine) or bidentate (bipyridine) ligand. The molecular preorganization of the bipyridine-based complex enables effective self-assembly in solution involving Pt⋯Pt interactions, while preserving aggregate solubility. On the other hand, increased steric effects of the linear bispyridine-based complex hinder an effective preorganization leading to poorly solvated aggregates when a critical concentration is exceeded.

## Abstract 2

The increase in preorganization associated with coordination geometry changes is exploited to regulate solvation effects and, in turn, processability of metallosupramolecular polymers exhibiting close metal–metal contacts.

## Graphical abstract

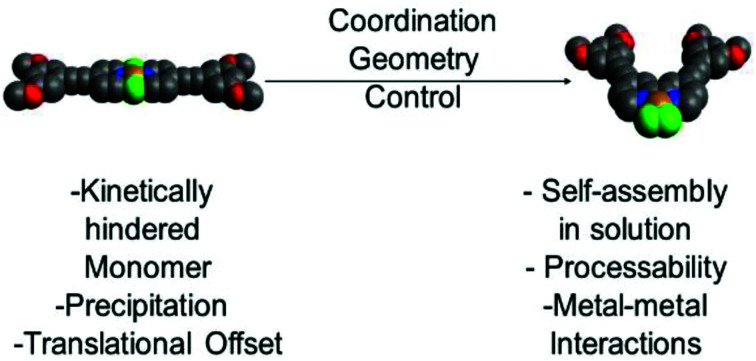

## Introduction

The desire to develop functional materials has helped kickstart numerous approaches to tune supramolecular self-assembly in solution.^1–5^ Particularly, to achieve precise properties, a constant balance between tailored aggregation, nanoscale morphology and processability has to be maintained.^6,7^ In this regard, living supramolecular polymerization represents a milestone towards controlling nanoscale morphology and supramolecular size distributions with defined aspect ratios.^8–13^ Similarly, secondary nucleation events at the outer sphere of a primary supramolecular assembly can be exploited to obtain a stepwise increase in size, or to acquire otherwise inaccessible morphologies.^14,15^ However, achieving similar dimensional control based on molecular design remains a challenging task towards application.

Dynamic supramolecular polymers with properties such as conductivity, self-healing or desired photophysical behaviour are prime candidates for the design of smart devices,^16,17^ as the bulk property is greater than in the monomeric state or self-assembly is a prerequisite for the emergence of certain properties.^18,19^ Nevertheless, some major challenges still hamper the progress of supramolecular polymers towards application, particularly in terms of finding the right balance between aggregation and solubility,^13,20,21^ which largely controls processability.^22,23^ Accordingly, design strategies that can attenuate initial steric hindrance and allow for a more controllable self-assembly process while preserving solubility and desired functional properties are a central goal in supramolecular chemistry.^24,25^

**Scheme 1:**
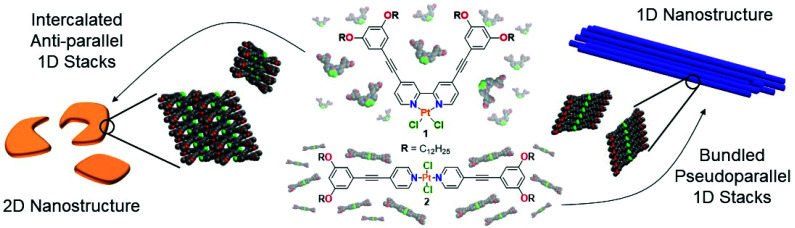
Molecular structures of **1** and **2** and schematic representation of their self-assembly in MCH.

Recently, we have demonstrated that the coordination geometry serves to control molecular preorganization events that, in turn, regulate the packing modes, energy landscapes and luminescence of metallosupramolecular polymers (Scheme S3†).^26^ However, nanoscale morphology and aggregation mechanism were influenced in a minor way. To further elucidate the influence of coordination and molecular geometry on self-assembly, we have modified our previous design to obtain complex **1** (Scheme 1). This chemical modification is expected to decrease the aggregation propensity and drive the system towards different pathways and molecular arrangements (see ESI† for expanded discussion). Further, a linear bispyridine-based complex **2** serves as reference compound, as the molecular composition remains identical besides the addition of formally two hydrogen atoms (Scheme 1). Based on our previous work, we hypothesize that the molecular preorganization exhibited by the V-shaped complex **1** should lead to a preferential self-assembly and improved solvation in comparison to **2**. This linear complex is expected to undergo a less controllable self-assembly into large superstructures due to the steric hindrance induced by the out-of-plane orientation of the chlorido ligands.

## Results and discussion

**Fig. 1:**
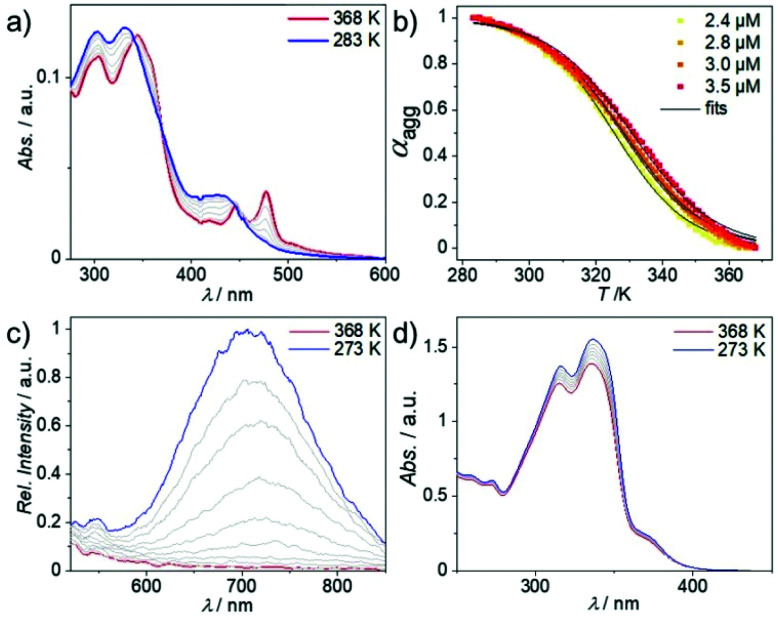
VT-UV/Vis (a) and VT-photoluminescence (c) of **1** at *c* = 3 × 10^−6^ M in MCH. (b) *α*_agg_ plotted against *T* fitted to the isodesmic model.^29^ (d) VT-UV/Vis of **2** at 3 × 10^−5^ M in MCH.

To examine the aggregation tendency of **1**, we initially used solvent-dependent UV/Vis studies at low concentration (*c* = 10 × 10^−6^ M, Fig. S9†). In accordance with previous reports on similar complexes, we identified low polarity solvents such as methylcyclohexane (MCH) as suitable candidates for aggregation studies. Subsequently, we turned to variable temperature (VT)-UV/Vis in MCH (*c* = 3 × 10^−6^ M) in order to get a better understanding of the self-assembly mechanism and preliminary information on the supramolecular packing mode (Fig. 1). **1** shows two lower energy absorption bands centered at 445 and 475 nm at high temperature (368 K), which can be attributed to a transition into a metal-to-ligand charge transfer state (MLCT, see Fig. S10† and subsequent discussion) of the molecularly dissolved state.^27^ Cooling to 283 K leads to the depletion of the absorption at 475 nm and a concurrent increase of a broader absorption between 400 and 450 nm with a maximum at 430 nm (Fig. 1a), pointing to the formation of *H*-type aggregates.^28^ Both high energy absorption bands show a similar hypsochromic shift of 5 and 15 nm, respectively. Plotting the degree of aggregation (*α*_agg_) calculated at *λ* = 475 nm *vs.* the temperature (*T*) shows a clear sigmoidal transition between the monomer and the aggregated state of **1** (an identical trend is observed in denaturation studies, see Fig. S11†). Consequently, the aggregation of **1** can be best described by the equal *K* (isodesmic) model^29^ (Fig. 1b, for detailed thermodynamic analysis see ESI; Fig. S12†) and the obtained fits accurately replicate the spectral data at various concentrations. These findings are in sharp contrast to the cooperative mechanism observed for a structurally related bipyridine-based Pt(ii) complex with a larger π-system.^26^ Thus, the reduction of the π-core leads to a switch from a cooperative to an isodesmic aggregation mechanism, which is also reflected in the absence of pathway complexity for **1**. These results point to a significant change in the intermolecular interactions governing the self-assembly process of both derivatives (*vide infra*). Additionally, we performed VT-photoluminescence studies of **1** using identical concentration, solvent and temperature range (Fig. 1c). During cooling from 368 K to 273 K, a broad emission between 600 and 850 nm progressively arises. Based on previous reports, we propose that this emission stems from the emergence of ^3^MMLCT states in combination with the decrease in non-radiative decay pathways caused by the temperature drop.^30^ Interestingly, the molecularly dissolved state does not show emissive behavior in the low energy region in a variety of good solvents, indicating that an aggregation-induced ^3^MMLCT excited state is observed. This is also supported by a more detailed solvent-dependent study (Fig. S14 and 15†). Subsequently, phosphorescence lifetime measurements of the aggregated form of **1** were performed after cooling a hot monomeric solution (*c* = 10 × 10^−6^ M, *T* = 368 K) to ambient conditions. Agg**1** shows an average lifetime of 116 ns (amplitude weighted) under degassed conditions, while further cooling to 77 K extends the long average lifetimes to roughly 14 μs (Fig. S16–S20†). These lifetimes in combination with the low energy featureless emission profile support our assignment to ^3^MMLCT states.

Comparing molecularly preorganized **1** with the linear compound **2** clearly shows the drastic influence of changing the coordination and molecular geometry. Solvent-dependent UV/Vis spectra of **2** disclose solvatochromic effects on the absorption profile. **2** exhibits only one broad absorption band at 330 nm in moderately polar solvents (chloroform and DCM; Fig. S21†), whereas decreasing the solvent polarity to *n*-hexane or MCH leads to a splitting of the broad absorption band into two bands at 315 and 335 nm (Fig. S22, for further details, see the ESI†). Concomitantly, a shoulder at 375 nm rises upon polarity decrease. VT-UV/Vis and VT photoluminescence studies reveal no signs of aggregation apart from a minor increase in absorption or emission (Fig. 1d, S23 and 24†), which can be assigned to the planarization of the aromatic system in the molecularly dissolved state upon cooling.^31^ This hypothesis is supported by the observation of well-defined sharp signals and only minor shifts in solvent-dependent ^1^H NMR spectra even at high MCH-d_14_ contents in CDCl_3_ (Fig. S25†), which is reaffirmed by VT-^1^H NMR in low polarity solvents (Fig. S26 and 27†). These results again highlight the significance of coordination geometry, as the linear compound **2** cannot effectively self-assemble in contrast to a previously reported compound with a larger aromatic surface,^26^ whereas **1** engages in a new aggregation pathway.

**Fig. 2:**
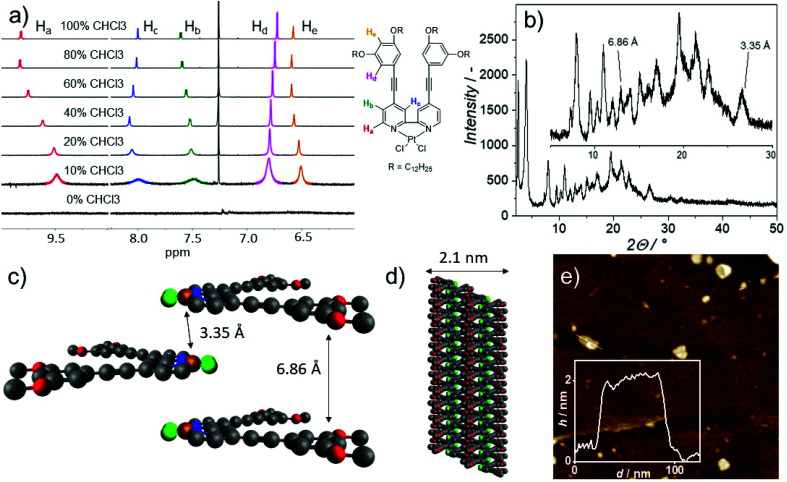
(a) Partial ^1^H NMR spectra of **1** in MCH-d_14_/CDCl_3_ mixtures (*c* = 1 × 10^−3^ M, *T* = 298 K) along with proton assignment. (b) PXRD diffraction pattern of Agg**1**, sample prepared from a solution in CHCl_3_/MCH (2 : 8, *c* = 7.5 × 10^−3^ M). (c and d) Schematic representation of the proposed packing mode of **1** in one (c) and two dimensions (d). (e) AFM height image of Agg**1**, inset: height profile.

Subsequently, we employed solvent- as well as temperature-dependent ^1^H NMR spectroscopy in order to understand the influence of coordination geometry and molecular preorganization on the packing mode of **1**. The stepwise increase of the volume fraction of MCH-d_14_ in CDCl_3_ leads to the aggregation of **1** at ambient conditions, which was also confirmed by solvent-dependent UV/Vis (Fig. 2a). The signals corresponding to the alpha proton H_a_ of the bipyridine exhibit the most significant upfield shift from 9.82 to 9.48 ppm, indicating their involvement in aromatic interactions.^32,33^ Further, the signal corresponding to the adjacent proton H_b_ exhibits only a very minor upfield shift (7.60 to 7.49 ppm), indicating weaker intermolecular interactions. The remaining signal corresponding to the bipyridine moiety (H_c_), however, shows a different behavior. Initially, up to 60% MCH-d_14_ in CDCl_3_, a downfield shift can be observed indicating the proximity of an electron rich group (such as O or Cl).^34^ At MCH fractions above 60%, a second process sets in, namely a minor upfield shift accompanied with significant broadening. The two proton signals corresponding to the peripheral phenyl rings exhibit a similar behavior, namely a downfield shift at high chloroform contents, followed by signal broadening upon increasing the MCH content. Moreover, it can be observed that the signals corresponding to the bipyridine moiety show much greater signal broadening compared to the signals of the phenyl ring. VT ^1^H NMR at intermediate solvent mixtures confirms this behavior (Fig. S28 and 29†). Moreover, ROESY NMR (see ESI for detailed discussion, Fig. S30†) suggests close intermolecular contacts between the alkyl chains and the aromatic protons of the neighboring bipyridine moiety. In combination with the intermolecular close contact between the OCH_2_ methylene unit and the alpha proton of the central bipyridine, we infer an antiparallel arrangement of the molecules within a 1D stack with possible interdigitation yielding 2D architectures. This proposed packing mode was further investigated by powder X-ray diffraction (PXRD). Numerous sharp reflexes can be observed correlating to distances between 4.5 and 10 Å. In particular a single reflex at 2*Θ* = 26.5° corresponding to a distance of 3.35 Å can be observed (Fig. 2b). In accordance with the observed lifetimes, we attribute this reflex to close Pt⋯Pt contacts, which lead to ^3^MMLCT excited states. However, no second reflex within a similar range can be observed, which suggests that the molecules do not stack in a parallel fashion, otherwise the π–π distance should also be observable.^35^ Hence, the distance between the aromatic units should be among the reflexes between 10 and 5 Å. In fact, the observation of a reflex at 6.86 Å, which we tentatively assign to the distance between aromatic moieties, supports this hypothesis. The proposed antiparallel arrangement further offers a plethora of diagonal spacings, which could explain the remaining reflexes. Combining the results from NMR and PXRD, we propose a model depicting the intermolecular packing (Fig. 2c and Fig. S31, 32†). The monomers of **1** stack in an antiparallel fashion, with the bipyridine moieties constituting the inner core of the 1D stacks leading to close intermolecular Pt⋯Pt contacts (Fig. 2d), in good agreement with the observed ^3^MMLCT emission (*vide supra*). The alkyl chains point towards the outside of the stack, effectively shielding the aromatic core from the nonpolar solvent. Additionally, this model offers a reasonable explanation for the more pronounced signal broadening of the bipyridine signals (H_a,b,c_) compared to the phenyl signals (H_d,e_) observed in solvent-dependent NMR, as the central part of the stack is more significantly involved in intermolecular interactions. This effective rearrangement in comparison to our previously reported compound^26^ clearly reflects that different coordination geometries can allow molecular entities to engage in new aggregation pathways balancing attractive intermolecular interaction and steric demand, which may not be the case for all ligand systems (*vide infra*).

In order to correlate the packing motif with the nanoscale morphology, we turned to atomic force microscopy (Fig. 2e and Fig. S33–35†). The aggregates of **1** can be best described as small 2D plates with a homogenous height of 2.1 nm, which matches well with the alignment of multiple 1D stacks. Additionally, keeping a highly concentrated solution of **1** in nonpolar solvents over time leads to the precipitation of microcrystalline assemblies consisting of smaller 2D plates (Fig. S36–38†). We speculate that this hierarchical level in the self-assembly behavior is the result of further stack intercalation leading to a 3D growth.

**Fig. 3:**
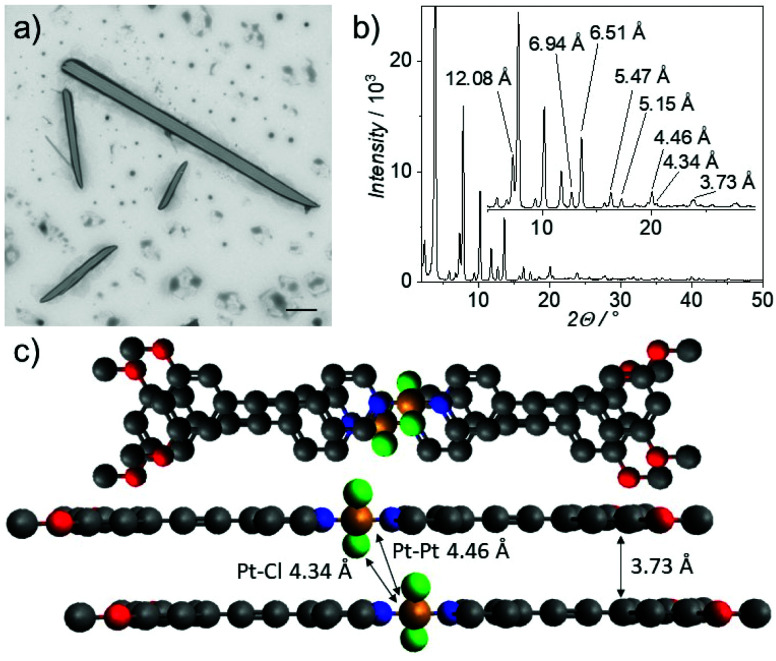
(a) SEM image of Agg**2**, the scale bar corresponds to 10 μm. (b) PXRD diffraction pattern of Agg**2**. Sample prepared from a solution in MCH (*c* = 1 × 10^−3^ M). (c) Representation of the proposed packing mode of Agg**2**.

Similarly, keeping highly concentrated solutions of **2** (*c* >1 mM) in solvents of low polarity (MCH content >80%, Fig. S36†) also leads to the formation of large needles with lengths between 10 and 200 microns and widths between 1 and 10 microns, as visualized by scanning electron microscopy (SEM, Fig. 3a and S39†). Upon closer inspection, it becomes apparent that these large structures consist of smaller fibers, which bundle together yielding larger morphologies (Fig. S40†). The microcrystalline nature of the needle-like structures is reflected in the well-defined diffraction pattern observed in PXRD (Fig. 3b). Comparing this pattern with molecular structures in the crystal of structurally related bispyridyldichlorido Pt(ii) complexes,^36^ we derive an almost parallel molecular packing with a small translational displacement to alleviate steric constraints (see Fig. S41† and subsequent discussion). These results highlight that modifying the coordination geometry to achieve molecular preorganization and alleviate steric constrains can enable spatiotemporal control over self-assembly and precipitation effects.

## Conclusions

In conclusion, we have analysed the role of coordination and molecular geometry in controlling the dimensionality and processability of self-assembled structures. To this end, we have compared the self-assembly behaviour of two Pt(ii) complexes with a different degree of preorganization (molecularly preorganized bipyridine-based **1** and non-preorganized bispyridine-based **2**) in nonpolar media. **1** self-assembles *via* the isodesmic mechanism into antiparallel 1D stacks that are stabilized by metal–metal interactions. Lateral interchain interactions between the stacks induce a hierarchical growth into solvated 2D plates and, subsequently to 2D microcrystalline plates. In sharp contrast, non-preorganized **2** does not self-assemble into solvated aggregates in solution. Instead, increasing the concentration in nonpolar media induces a sudden self-assembly process that leads to rapid precipitation of large bundles of fibers. Our results disclose coordination geometry as an effective tool to achieve dimensional control and processability, which is a prerequisite for the design and application of functional nanomaterials.

## Author contributions

The manuscript was written through contribution of all authors. All authors have given approval to the final version of the manuscript.

## Conflicts of interest

There are no conflicts to declare.

## Supplementary Material

QO-008-D1QO00644D-s001
