# Supplementary material for: Exploiting coordination geometry to tune the dimensions and processability of metallosupramolecular polymers
Source: Org Chem Front. 2021 Jun 4;8(15):4138–43. doi: 10.1039/d1qo00644d (PMC8314868; doi:10.1039/d1qo00644d)
Supplement: QO-008-D1QO00644D-s001 [file QO-008-D1QO00644D-s001.pdf]

Electronic Supplementary Material (ESI) for Organic Chemistry Frontiers.
This journal is © the Partner Organisations 2021

Electronic Supplementary Information

Exploiting Coordination Geometry to tune the Dimensions and Processability of Metallosupramolecular Polymers

Nils Bäumer,[a] Kalathil K. Kartha,[a] Stefan Buss,[b] Jasnamol P. Palakkal,[c] Cristian A. Strassert,[b] Gustavo Fernández[a,*]

[a]*Organisch-Chemisches Institut, Universität Münster, Corrensstraße 36, 48149 Münster, Germany.*

[b]*Institut für Anorganische und Analytische Chemie, CiMIC, SoN, Westfälische Wilhelms-Universität Münster, Corrensstraße 28/30, 48149 Münster, Germany; CeNTech, Westfälische Wilhelms-Universität Münster, Heisenbergstraße 11, 48149 Münster, Germany.*

[c]*Institute of Materials Science, Technische Universität Darmstadt, 64287 Darmstadt, Germany.*

Table of contents

Materials and Methods 3
Synthesis and Characterization 5
Additional Spectroscopic Data 12
References 26

Materials and Methods

General Procedures: All solvents were dried according to standard procedures. Reagents were used as purchased. All air-sensitive reactions were carried out under argon atmosphere.

NMR measurements: ^{1}H and ^{13}C NMR spectra were recorded on a Bruker Avance 400 (^{1}H: 400 MHz; ^{13}C: 100.6 MHz) and a Bruker AV300 (^{1}H: 300 MHz; ^{13}C: 100.6 MHz). Additional 1D ^{1}H as well as 2D ^{1}H- ^{1}H COSY and 2D ^{1}H- ^{1}H ROESY spectra were recorded on an Agilent DD2 500 (^{1}H: 500 MHz) and an Agilent DD2 600 (^{1}H: 600 MHz) at a standard temperature of 298 K in deuterated solvents. Deviating temperature is explicitly mentioned when used. The recorded spectra were referenced to the remaining resonance signals of the deuterated solvents ($CDCl_3$: 7.26 ppm (^{1}H); DCM: 5.32 ppm (^{1}H)). The coupling constant J of the measured spin multiplets is given in Hertz (Hz) and the chemical shifts are given in reference to the chemical shift of trimethylsilane (0 ppm). The abbreviations used to analyze the recorded spectra are: s (singlet), d (doublet), m (multiplet).

Mass spectroscopy: MALDI-mass spectra were recorded on an Autoflex Speed manufactured by Bruker Daltronics. A SmartBeamTM NdYAF-Laser with a wavelength of 335 nm was used. The signals are described by their mass/charge ratio (m/z) in u.

UV-Vis spectroscopy: All UV-Vis spectra were recorded on a V-770 and a V-750 by the company JASCO with a spectral bandwidth of 1.0 nm and a scan rate of 400 nm min^{-1}. Glass cuvettes with an optical length of 1 cm and 1 mm were used. All measurements have been conducted in solvents from commercial sources of spectroscopic grade.

Atomic force microscopy: The AFM images have been recorded on a Multimode®8 SPM Systems manufactured by Bruker AXS. The used cantilevers were AC200TS by Oxford Instruments with an average spring constant of 9 N m^{-1}, an average frequency of 150 kHz, an average length of 200 µm, an average width of 40 µm and an average tip radius of 7 nm. All solutions have been spin-coated onto an HOPG surface using a spin rate between 2000 and 4000 rpm.

Steady-state photoluminescence spectroscopy: All emission spectra were recorded on a JASCO FP-8500 spectrofluorometer. Glass cuvettes with an optical length of 1 cm were used. All measurements have been conducted in solvents from commercial sources of spectroscopic grade.

Lifetime measurements: Steady-state excitation and emission spectra were recorded on a FluoTime300 spectrometer from PicoQuant equipped with a 300 W ozone-free Xe lamp (250-900 nm), a 10 W Xe flash-lamp (250-900 nm, pulse width < 10 µs) with repetition rates of 0.1 – 300 Hz, an excitation monochromator (Czerny-Turner 2.7 nm/mm dispersion, 1200 grooves/mm, blazed at 300 nm), diode lasers (pulse width < 80 ps) operated by a computer-controlled laser driver PDL-820 (repetition rate up to 80 MHz, burst mode for slow and weak decays), two emission monochromators (Czerny-Turner, selectable gratings blazed at 500 nm with 2.7 nm/mm dispersion and 1200 grooves/mm, or blazed at 1250 nm with 5.4 nm/mm dispersion and 600 grooves/mm), Glan-Thompson polarizers for excitation (Xe-lamps) and emission, a Peltier-thermostatized sample holder from Quantum Northwest (-40 °C – 105 °C), and two detectors, namely a PMA Hybrid 40 (transit time spread FWHM < 120 ps, 300 – 720 nm) and a R5509-42 NIR-photomultiplier tube (transit time spread FWHM 1.5 ns, 300-1400 nm) with external cooling (-80 °C) from Hamamatsu. Steady-state emission spectra and lifetimes were recorded in TCSPC mode by a PicoHarp 300 (minimum base resolution 4 ps). Phosphorescence lifetimes were recorded in MCS mode by a NanoHarp 250 (minimum base resolution 4 ns). Emission and excitation spectra were corrected for source intensity (lamp and grating) by standard correction curves. Lifetime analysis was performed using the commercial FluoFit software. The quality of the fit was assessed by minimizing the reduced chi squared function (χ2) and visual inspection of the weighted residuals and their autocorrelation. Luminescence quantum yields were measured with a Hamamatsu Photonics absolute PL quantum yield measurement system (C9920-02) equipped with a L9799-01 CW Xenon light source (150 W), monochromator, C7473 photonic multi-channel

analyzer, integrating sphere and employing U6039-05 PLQY measurement software (Hamamatsu Photonics, Ltd., Shizuoka, Japan). All samples were measured in Suprasil® quartz cuvettes with septa. Deaerated samples were measured upon flushing with argon for 20 min (using the septa with a cannula).

X-ray diffractometric analysis: Powder X-ray diffraction experiments were conducted on a ®Rigaku Smartlab X-ray diffractometer in parallel beam geometry with Cu-Kα radiation. The measurement steps were 0.01 deg with a measurement time per step of 0.6 s, using a voltage of 45 kV and a current of 190 mA. Samples have been prepared by stepwise dropcasting a solution with a concentration of 7.5 mM (MCH) onto a glass microscope plate from FisherbrandTM with a thickness of 0.8-1 mm.

Scanning Electron Microscopy: The SEM images were recorded on a Phenom Pharos Desktop SEM and on a Phenom ProX Desktop SEM manufactured by Thermo Fisher Scientific. The individual images have been recorded using a zoom between 22500x and 300x with either a BSD or SED detector and an acceleration voltage of either 5 or 10 kV (For individual images please see the corresponding figure caption). All solutions have been dropcasted onto a silicon wafer (10 µL) and additionally coated with Pt using a physical vapor deposition for a time interval of 30 seconds prior to measurement.

Synthesis and Characterization

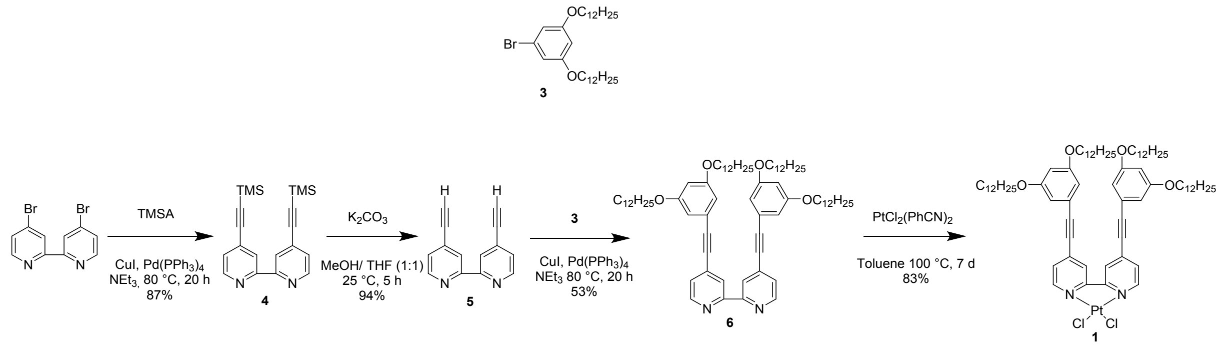

Scheme S1: Synthesis of complex **1**.

Compound **3** was synthesized according to procedures previously reported by our group.[1,2]

Synthesis of **4**

4 was synthesized according to a previously reported method.[3] 4,4'-Dibromo-2,2'-bipyridine (500 mg, 1.59 mmol, 1.00 eq.), $Pd(PPh_3)_4$ (91.3 mg, 0.08 mmol, 0.05 eq.) and CuI (3 mg, 0.02 mmol, 0.01 eq.) were dissolved in degassed NEt_3 (25 ml) and stirred at room temperature for 20 min. Afterwards, TMSA (375 g, 3.82 mmol, 2.40 eq.) was added dropwise. The solution was stirred at 80 °C for 20 h. After the reaction was complete, the solvent was evaporated and the crude product purified by column chromatography (SiO_2, DCM/ MeOH (98:2)). The product was obtained as a white powder.

Yield: 483 mg, 1.38 mmol, 87%

^{1}H-NMR (300 MHz, $CDCl_3$, 298 K): δ = 8.65-8.58 (m, 2H); 8.45-8.41 (m, 2H); 7.35-7.30 (m, 2H); 0.26 (s, 18 H) ppm.

Synthesis of **5**

5 was synthesized according to a previously reported method.[3] **4** (530 mg, 1.53 mmol, 1.00 eq.) and K_2CO_3 (1.00 g, 7.27 mmol, 4.76 eq.) were dissolved in a mixture of MeOH and THF (40 mL, 1:1). The solution was stirred at 25 °C for 5 h. A mixture of water and DCM (40 mL, 1:1) was added. The organic phases were separated, washed with water (3 ×15 mL) and brine (15 mL) and dried over $MgSO_4$. The solvent was removed *in vacuo* and the product obtained as a yellow powder.

Yield: 294 mg, 1.44 mmol, 94%

^{1}H-NMR (300 MHz, $CDCl_3$, 298 K): δ = 8.66 (d, *J* = 5.0 Hz, 1H); 8.48 (s, 1H), 7.38 (d, *J* = 5.0 Hz, 1H); 3.32 (s, 1H) ppm.

Synthesis of **6**

5 (244 mg, 1.19 mmol, 1 eq.), **3** (1.51 g, 2.87 mmol, 2.40 eq.), $Pd(PPh_3)_4$ (70 mg, 0.059 mmol, 0.050 eq.) and CuI (2 mg, 0.012 mmol, 0.010 eq.) were dissolved in degassed NEt_3 (25 mL) and stirred at 25 °C for 30 min. Afterwards, the solution was stirred at 80 °C for 20 h. The solvent was removed *in vacuo*. The crude product was purified using column chromatography (SiO, DCM/pentane (1:1)) yielding the product as a white solid.

Yield: 644 mg, 0.63 mmol, 53%

^{1}H-NMR (600 MHz, $CDCl_3$, 298 K): δ = 8.68 (d, J = 5.0 Hz, 2H); 8.56 (s, 2H); 7.42 (d, J = 5.0, 2H); 6.70 (d, J = 2.3 Hz, 4H); 6.51 (t, J = 2.3 Hz, 2H); 3.96 (t, J = 6.6 Hz, 8H), 1.82-1.75 (m, 8H); 1.49-1.42 (m, 8H); 1.38-1.34 (m, 8H); 1.33-1.24 (m, 56H); 0.88 (t, J = 7.0 Hz, 12H) ppm.

^{13}C-NMR (600 MHz, $CDCl_3$, 298 K): δ = 160.30, 155.35, 149.16, 133.01, 125.77, 123.64, 123.33, 110.29, 103.88, 94.90, 86.36, 68.42, 32.07, 29.82, 29.79, 29.76, 29.74, 29.52, 29.50, 29.33, 26.17, 22.84, 14.26 ppm.

HRMS (ESI - in MeOH): m/z calculated for $[M+H]^+$ = 1093.86991; found = 1093.86949.

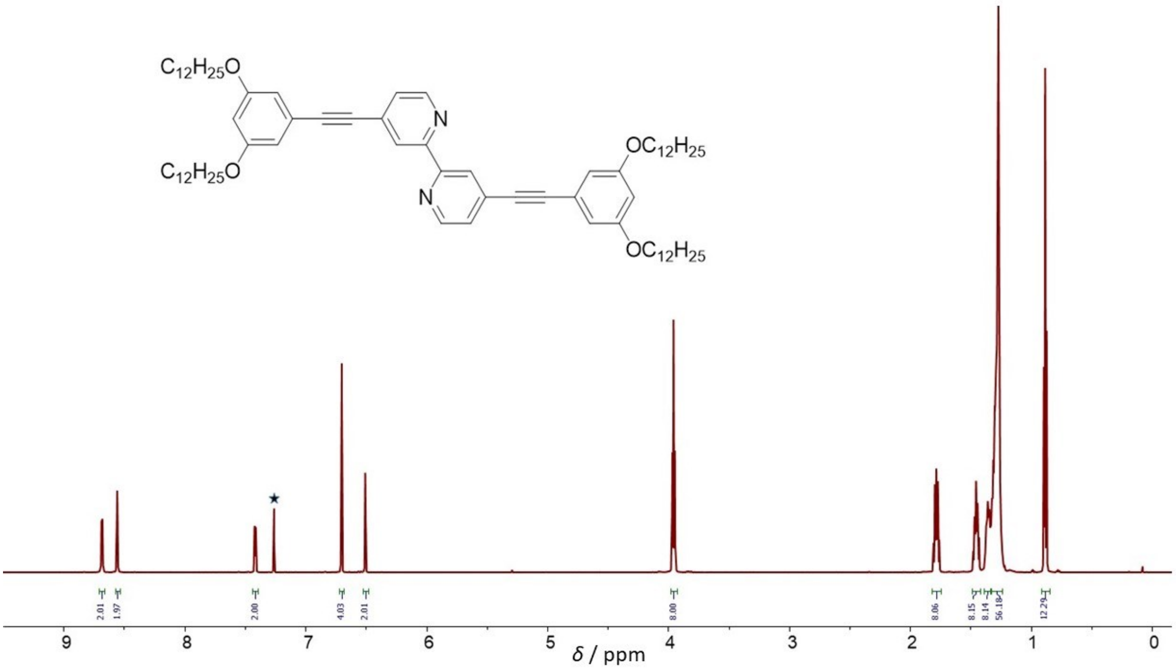

Figure S1: ^{1}H NMR spectrum of **6** (400 MHz, $CDCl_3$, 298 K).

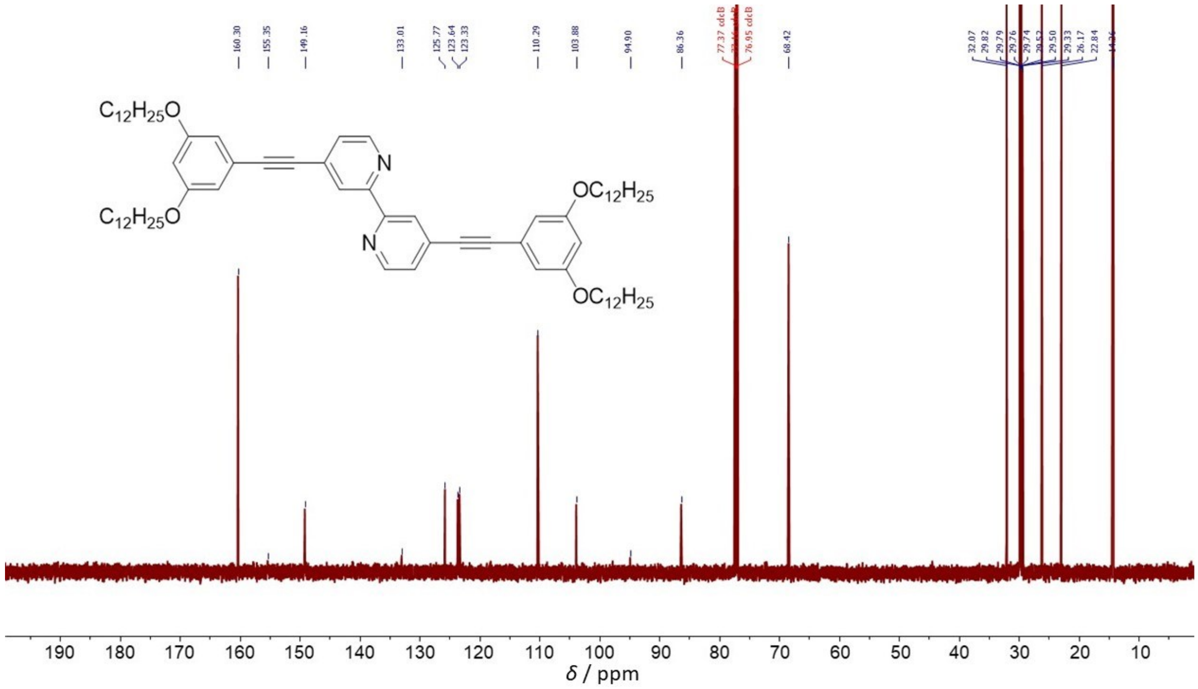

Figure S2: ^{13}C NMR spectrum of **6** (100.6 MHz, $CDCl_3$, 298 K).

Synthesis of **1**

6 (40 mg, 0.037 mmol, 1.0 eq.) and $PtCl_2(PhCN)_2$ (17 mg, 0.037 mmol, 1.0 eq.) were dissolved in distilled toluene (5 mL) under Argon and stirred at 100 °C for 7 days. The solvent was removed *in vacuo* and the crude product was purified by column chromatography (SiO_2, DCM) to give an orange powder.

Yield: 42 mg, 0.031 mmol, 83%

^{1}H-NMR (400 MHz, $CDCl_3$, 298 K): δ = 9.52 (d, J = 6.1 Hz, 2H); 8.01 (s, 2H); 7.50 (d, J = 6.1 Hz, 2H); 6.70 (d, J =2.2 Hz, 4H); 6.50 (t J = 2.2 Hz, 2H); 3.94 (t, J = 6.6 Hz, 8H); 1.79-1.73 (m, 8H); 1.46-1.38 (m, 8H); 1.35-1.30 (m, 8H); 1.30-1.24 (m, 64H); 0.88 (t, J = 6.7 Hz, 12H) ppm.

^{13}C-NMR (400 MHz, $CDCl_3$, 298 K): δ = 160.36, 156.37, 148.95, 135.00, 128.80, 125.21, 122.15, 110.51, 104.89, 100.69, 85.39, 68.55, 32.07, 29.84, 29.81, 29.80, 29.76, 29.60, 29.51, 29.38, 26.19, 22.84, 14.26 ppm.

HRMS (ESI in MeOH/$CHCl_3$): m/z calculated 1381.75564 $[M+Na]^+$; found 1381.75439.

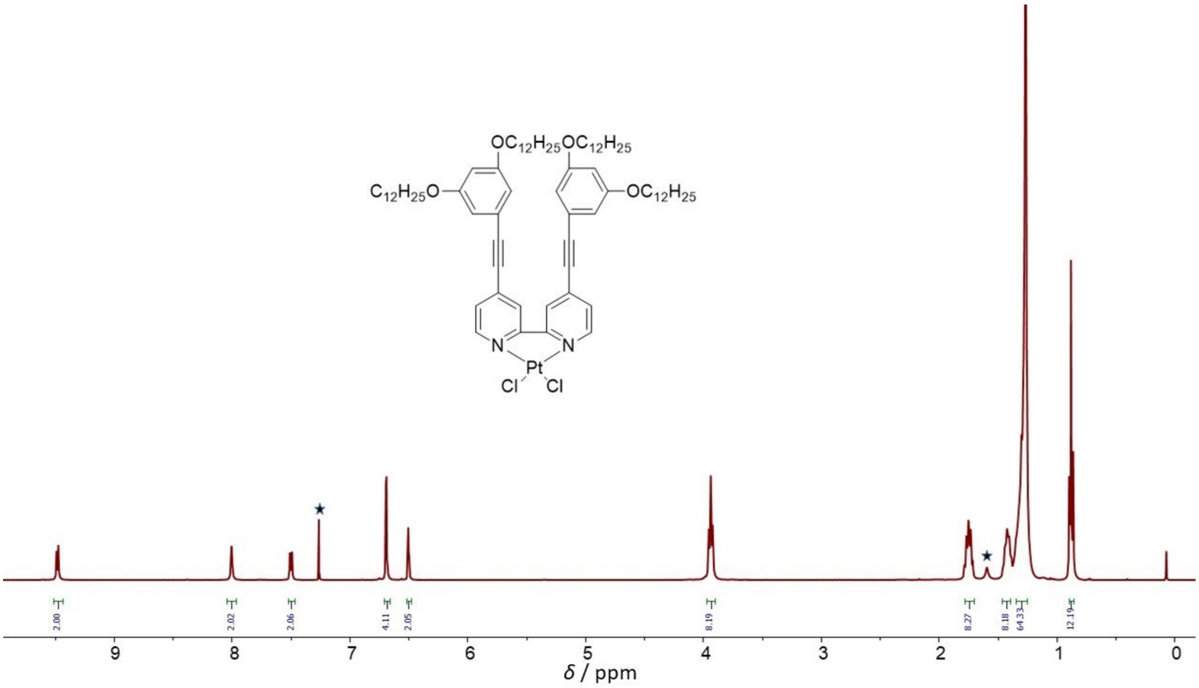

Figure S3: ^{1}H NMR spectrum of **1** (400 MHz, $CDCl_3$, 298 K).

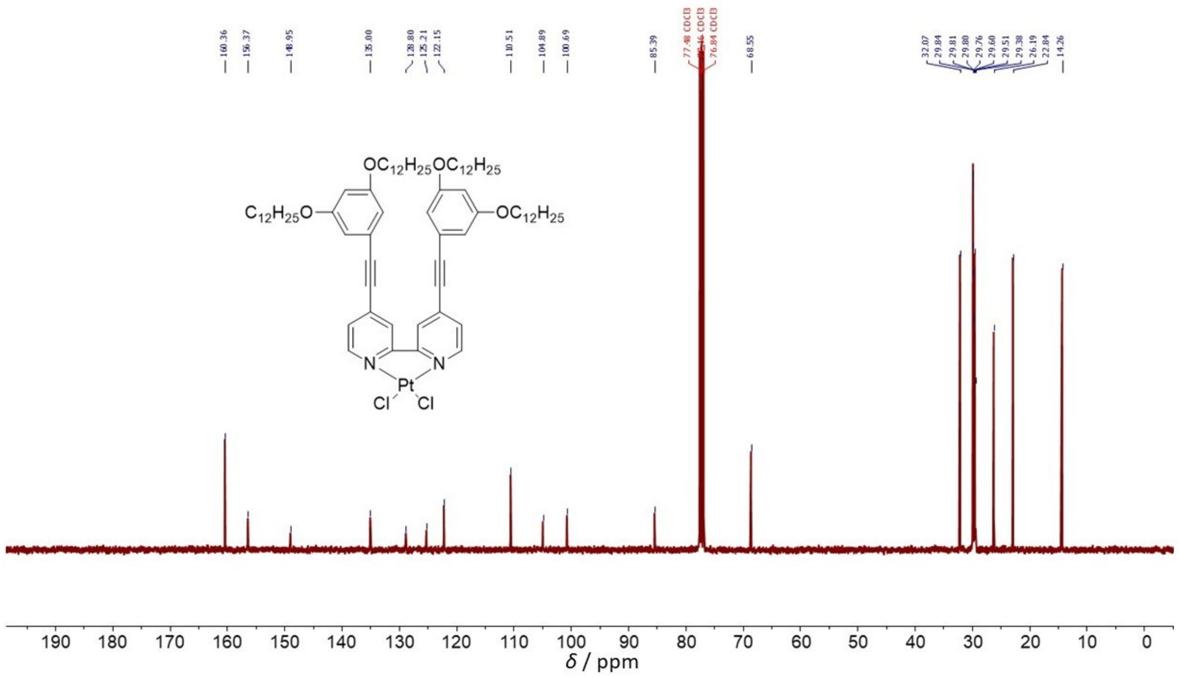

Figure S4: ^{13}C NMR spectrum of **1** (100.6 MHz, $CDCl_3$, 298 K).

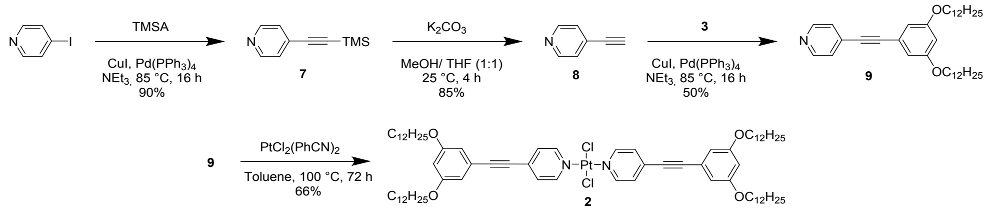

Scheme S2: Synthesis of complex **2**.

Synthesis of **7**

7 was synthesized according to a previously reported procedure.[4] $Pd(PPh_3)_4$ (173.0 mg, 0.15 mmol, 0.06 eq.), CuI (17.0 mg, 0.09 mmol, 0.04 eq.) and 4-Iodopyridine (614.8 mg, 2.99 mmol, 1.19 eq.) were dissolved in freshly degassed NEt_3 (20 mL) under argon atmosphere and stirred for 20 min at room temperature. Subsequently, TMSA (350 µL, 248 mg, 2.52 mmol, 1.00 eq.) was added dropwise and the solution was stirred at 85 °C for 16 h. The solvent was evaporated and the crude product was purified by column chromatography (SiO_2, pentane/DCM 50:50). The product was obtained as a white powder.

Yield: 473 mg, 2.70 mmol, 90%

^1H-NMR (300 MHz, $CDCl_3$, 298 K): δ = 8.59 (d, J = 6.1 Hz, 2H); 7.34 (d, J = 6.1 Hz, 2H); 0.31 (s, 9H) ppm.

Synthesis of **8**

8 was synthesized according to a previously reported procedure.[4] **7** (134.6 mg, 0.77 mmol, 1.00 eq.) and K_2CO_3 (265 mg, 1.90 mmol, 2.50 eq.) were dissolved in a mixture of THF and methanol (30 mL, 1:1). The solution was stirred at room temperature for 4 h. DCM and water were added (20 mL, 1:1). The phases were separated and the aqueous phase was extracted with DCM (3 · 10 mL). The combined organic phases were dried over $MgSO_4$ and the solvent was removed *in vacuo*. The crude product was purified by column chromatography (SiO_2, DCM/methanol 99:1), yielding a white powder.

Yield: 67.5 mg, 0.65 mmol, 85%.

^{1}H-NMR (300 MHz, $CDCl_3$, 298 K): δ = 8.56 (d, J = 6.0 Hz, 2H); 7.30 (d, J = 6.0 Hz, 2H); 4.95 (s, 1H).

Synthesis of **9**

$Pd(PPh_3)_4$ (50 mg, 0.04 mmol, 0.05 eq.), CuI (5 mg, 0.02 mmol, 0.03 eq.) and **4** (460 mg, 0.88 mmol, 1.0 eq.) were dissolved in freshly degassed NEt_3 (10 mL) under argon atmosphere and stirred at room temperature for 30 min. Subsequently **9** (90 mg, 0.88 mmol, 1.0 eq.) was added and the mixture was stirred at 85 °C for 16 h. The solvent was removed *in vacuo* and the crude product was purified by column chromatography (SiO_2, DCM/ methanol 99:1). The product was obtained as a white powder.

Yield: 190 mg, 0.44 mmol, 50%.

^{1}H-NMR (400 MHz, $CDCl_3$, 298 K): δ = 8.59 (d, J = 5.1 Hz, 2H); 7.36 (d, J = 5.1 Hz, 2H); 6.67 (d, J = 2.3 Hz, 2H); 6.49 (t, J = 2.3 Hz, 1H); 3.94 (t, J = 6.5 Hz, 4H); 1.84 – 1.71 (m, 4H); 1.51 – 1.41 (m, 4H); 1.38 – 1.21 (m, 32H); 0.88 (t, J = 6.8 Hz, 6H) ppm.

^{13}C-NMR (400 MHz, $CDCl_3$, 298 K): δ = 160.17, 149.77, 131.43, 125.57, 123.13, 110.10, 103.54, 94.22, 85.93, 68.24, 31.93, 29.68, 29.65, 29.61, 29.59, 29.38, 29.36, 29.20, 26.03, 22.70, 14.13 ppm.

MicroTof-ESI (MeOH): m/z calculated for $[M+H]^+$ = 548.4389, found = 548.4447.

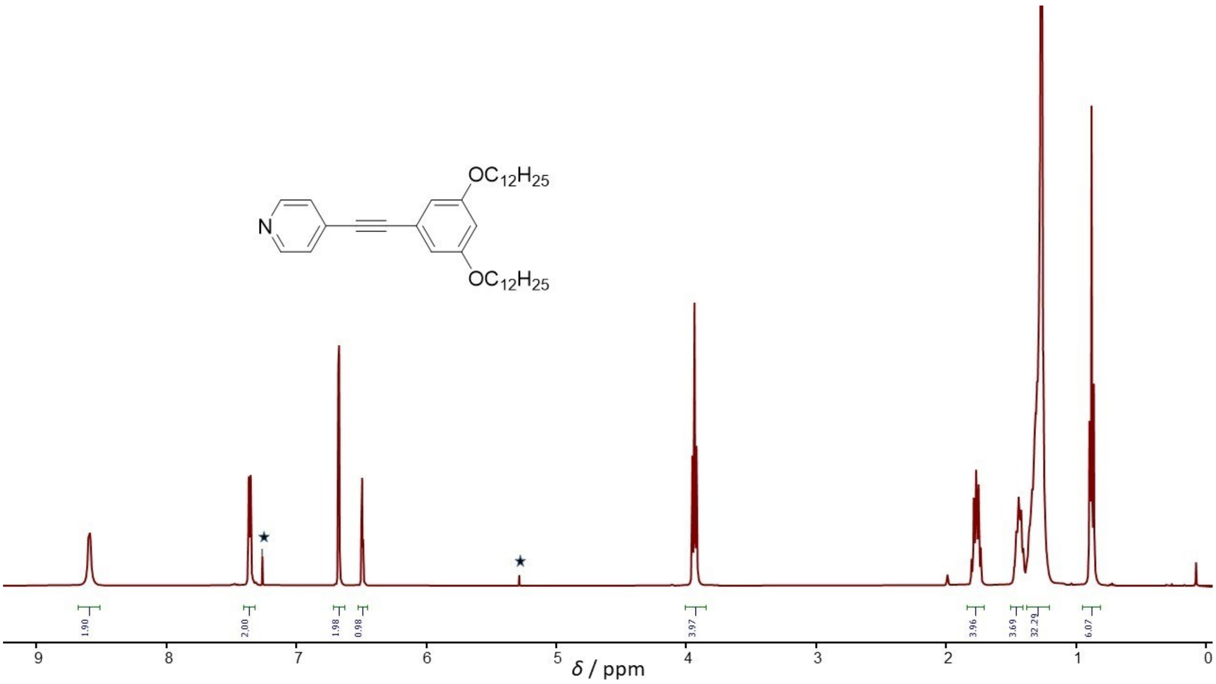

Figure S5: ^{1}H NMR spectrum of **9** (400 MHz, $CDCl_3$, 298 K).

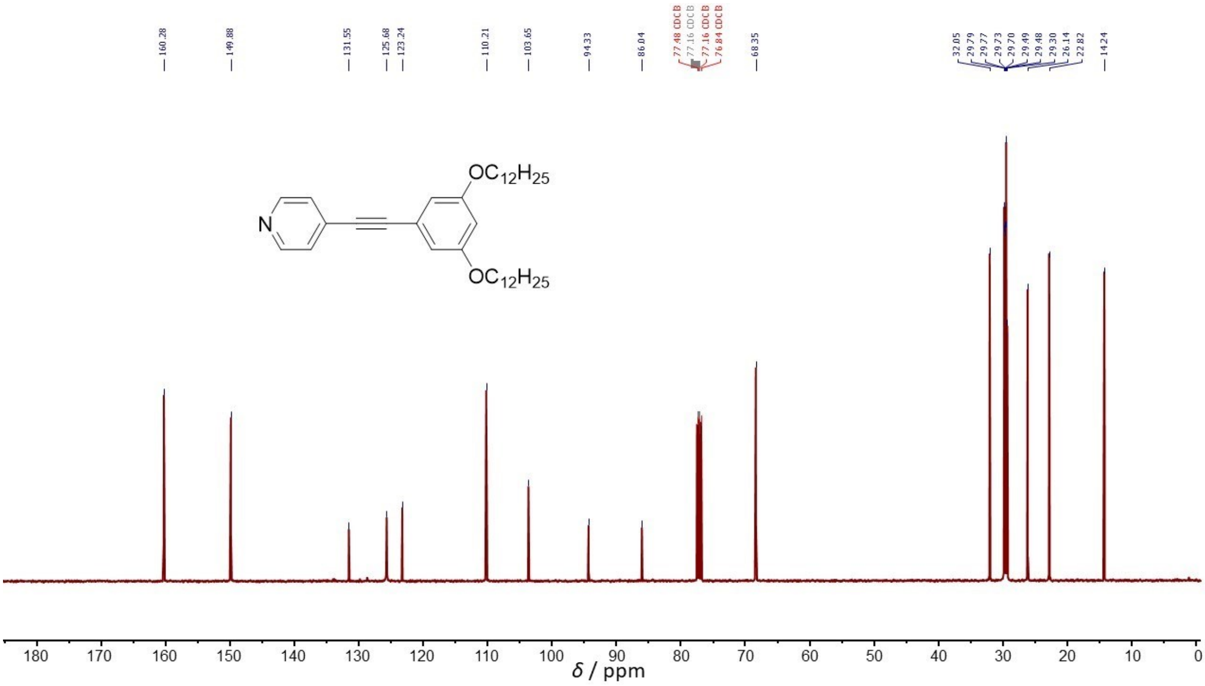

Figure S6: ^{13}C NMR spectrum of **9** (100.6 MHz, $CDCl_3$, 298 K).

Synthesis of **2**

9 (100 mg, 0.18 mmol, 2.0 eq.) and $PtCl_2(PhCN)_2$ (43 mg, 0.09 mmol, 1.0 eq.) were dissolved in dried toluene (10 mL) and stirred at 100 °C for 72 h. The solvent was removed and the crude product purified by column chromatography (SiO_2, pentane/DCM 50:50). Minor impurities that could not be removed by column chromatography could be removed by precipitation from DCM in diethylether. The product was obtained as pale-yellow powder.

Yield: 80 mg, 0.06 mmol, 66%.

^1H-NMR (400 MHz, $CDCl_3$, 298 K): δ = 8.86 (d, 7.0 Hz, 2H), 7.34 (d, *J* = 7.0 Hz, 2H), 6.68 (d, *J* = 2.3 Hz, 2H), 6.52 (t, *J* = 2.3 Hz, 1H), 3.94 (t, *J* = 6.5 Hz, 4H), 1.82 – 1.72 (m, 4H), 1.49 – 1.39 (m, 4H), 1.43 – 1.33 (m, 4H), 1.38 – 1.18 (m, 32H), 0.88 (t, *J* = 6.8 Hz, 6H) ppm.

^{13}C-NMR (400 MHz, $CDCl_3$, 298 K): δ = 160.22, 153.12, 134.24, 126.98, 122.28, 110.30, 104.28, 98.61, 84.71, 77.36, 77.04, 76.72, 68.31, 31.93, 29.68, 29.65, 29.61, 29.59, 29.45, 29.37, 29.17, 26.02, 22.71, 14.14 ppm.

HRMS (ESI in MeOH/$CHCl_3$): *m/z* calculated for $[M+Na]^+$ = 1383.77004; found = 1383.77362.

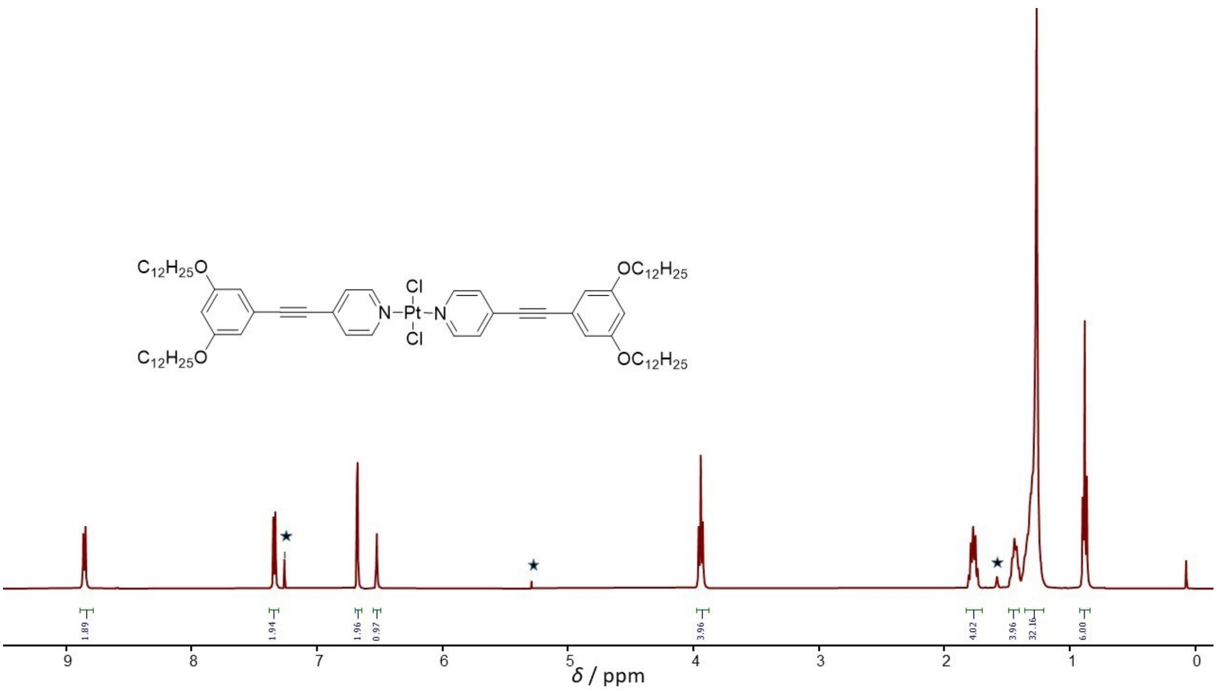

Figure S7: ^{1}H NMR spectrum of **2** (400 MHz, $CDCl_3$, 298 K).

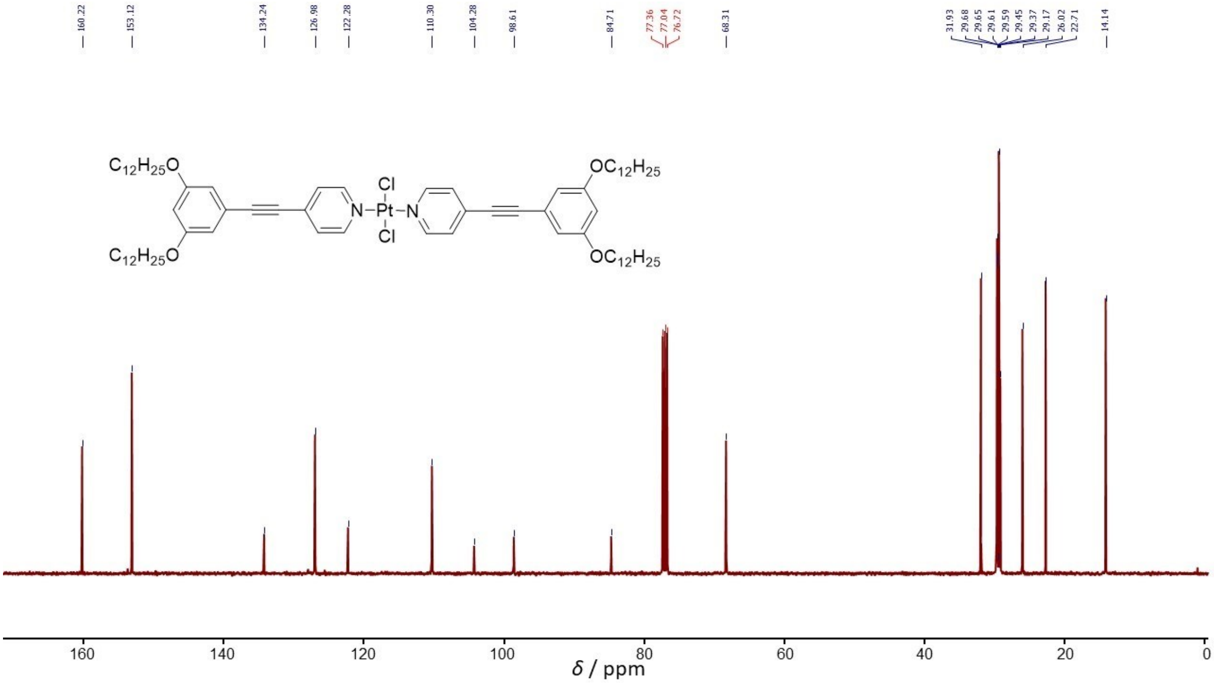

Figure S8: ^{13}C NMR spectrum of **2** (100.6 MHz, $CDCl_3$, 298 K).

Additional Spectroscopic Data

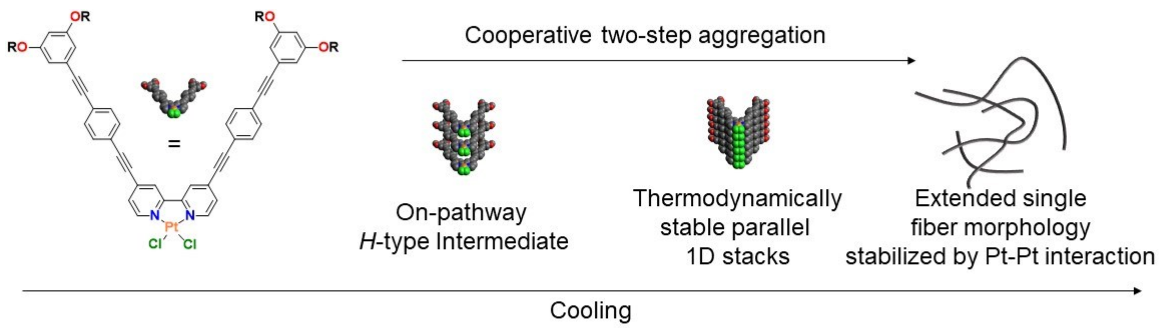

Scheme S3: Schematic representation of the self-assembly of a structurally related bipyridine based Pt^{II} complex with a larger aromatic OPE system.[2]

Our previous report was the first study[2] detailing the influence of coordination geometry on self-assembly. Accordingly, more examples based on a modified molecular design are necessary to understand the structure-property relationship in detail. Hence, we designed the molecules **1** and **2** in our current submission, which exhibit a smaller aromatic moiety and in turn a lower aggregation tendency. V-shaped **1** in our previous example exhibited pathway complexity in a two-step cooperative self-assembly process into a single aggregate (solvated fiber structures). We expected the change in intermolecular interactions in our design to alter this behavior, as the steric demand of the bulky metal center and the flexible solubilizing chains are now balanced against weaker aggregation-inducing interactions. Consequently, we anticipated the system to engage in a different self-assembly behavior to balance the now weaker aggregation inducing interactions by following new aggregation pathways or forming altered molecular arrangements, changing the overall morphology.

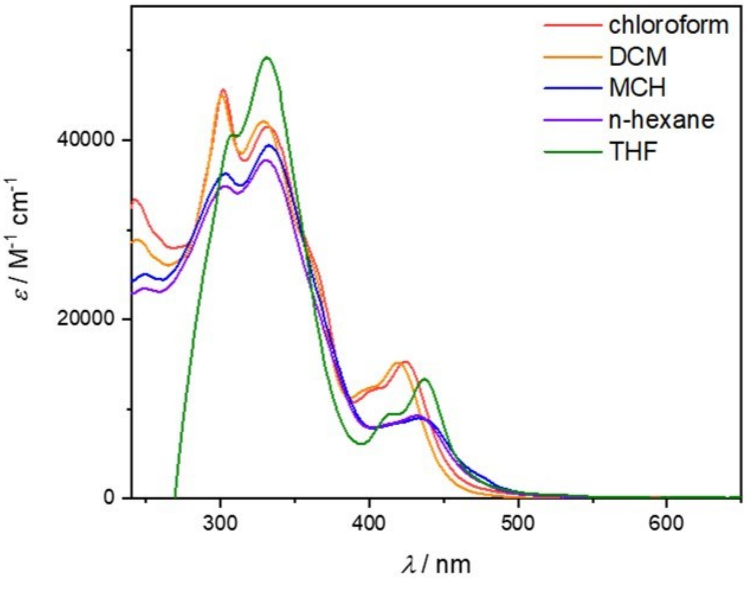

Figure S9: Solvent-dependent UV-Vis absorption spectra of **1** at c = 1 x 10^{-5} M and 298 K.

Complex **1** shows very similar absorption bands in chloroform/DCM and MCH/pentane, respectively. Two high energy absorption bands at 300 and 330 nm and two lower energy absorption bands (400 and 420 nm) are observed in more polar media, whereas only one absorption band in the lower energy region for less polar solvents (434 nm) can be observed. THF presents an intermediate behavior between the two spectra.

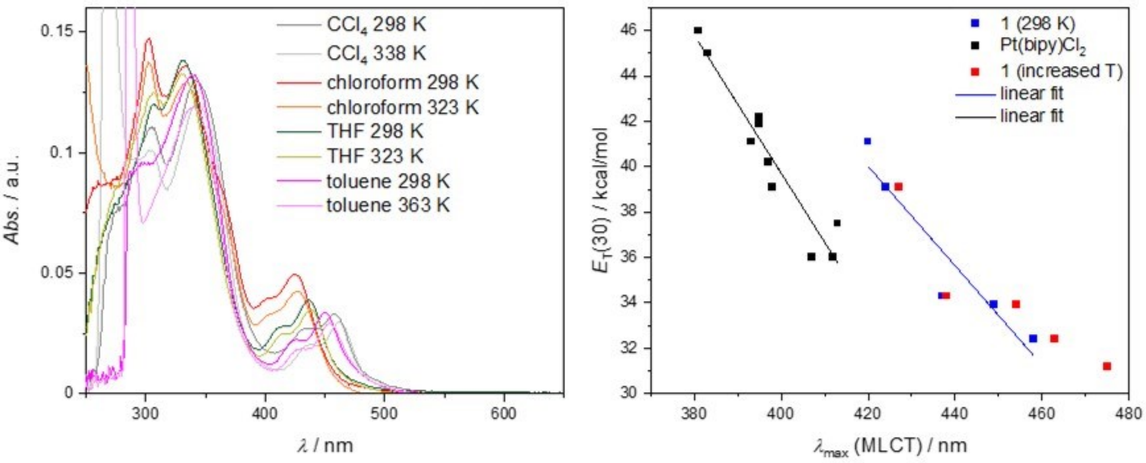

Figure S10: VT-solvent dependent UV-Vis absorption spectra of **1** at *c* = 1 x 10^{-5} M (left). Absorption maximum of the band assigned to the transition into the MLCT state plotted against the solvent polarity,[5] with a graphical comparison to structurally related compound $Pt(bipy)Cl_2$ confirming the assignment.[6]

In order to confirm the assignment of the low energy absorption band in MCH at high temperature, the absorption maximum was plotted against the solvent polarity in a number of solvents (Figure S9 + S10). A linear fit of the maximum absorbance against the solvent polarity conducted for **1** (at 298 K) is nearly parallel to that of previously reported $Pt(bipy)Cl_2$[6] with an offset corresponding to the shift in absorbance observed in the molecularly dissolved state in chloroform. Additionally, we recorded UV/Vis spectra at elevated temperatures revealing a temperature-dependent shift of the absorption maximum for all solvents. As the molecularly dissolved state in MCH cannot be recorded at 298 K due to aggregation, a comparison of the data recorded at elevated temperatures is more appropriate. Unfortunately, most organic solvents have a boiling point well below that of MCH (which is also the reason why it is frequently used in aggregation studies), one exception being toluene. In toluene, the absorbance at 363 K shifts to 454 nm (from 449 nm at room temperature), which indicates that a similar behavior would also be observed for MCH. Taking all these points into consideration, we conclude that the low energy absorbance should be assigned to MLCT transitions.

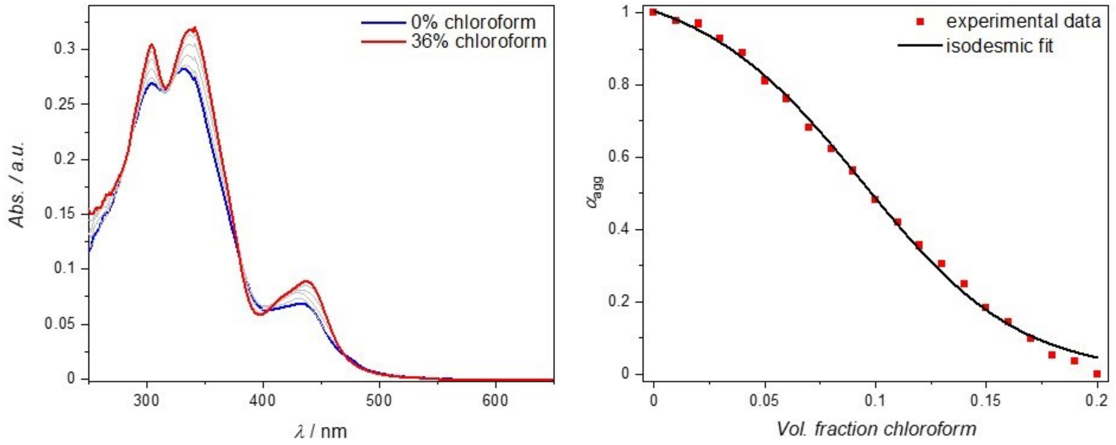

Figure S11: Solvent-dependent UV/Vis absorption spectra of **1** for different ratios of $CDCl_3$ and MCH-d_{14} at *c* = 1 x 10^{-5} M and *T* = 298 K, revealing an isodesmic denaturation behavior.

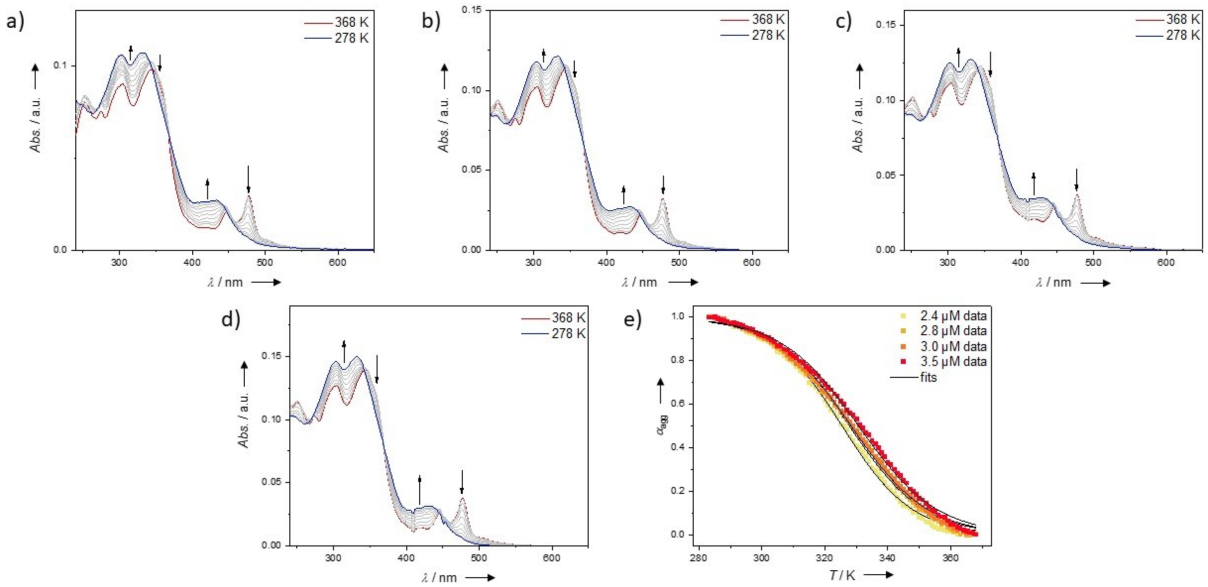

Figure S12: Variable temperature (VT)-UV-Vis spectra of **1** at *c* = 2.4 x 10^{-6} M (a); 2.8 x 10^{-6} M (b); 3.0 x 10^{-6} M (c); and 3.5 x 10^{-6} M (d) in MCH between 368 and 278 K with the degree of aggregation α_{agg} (calculated from the depletion of the absorbance at λ = 475 nm) plotted vs temperature (*T*) and fits were obtained from isodesmic model using a global fitting approach (e).[7]

Table S1: Thermodynamic parameters T_m; ΔH; ΔS; ΔG and *K* derived from VT-UV-Vis experiments of **1** (MCH, λ = 475 nm) on the basis of the equal K model using global fitting.[7] Note that the thermodynamic analysis was based on spectral changes of *ΔAbs.* = 0.05. Accordingly, the experimental uncertainty should be considered given as standard deviation in parenthesis (SD).

Concentration / 10^{-6} M	T_m (SD) / K	ΔH (SD) / kJ mol^{-1}	ΔS (SD) / kJ mol^{-1}	ΔG (SD) / kJ mol^{-1}	*K* (SD) / 10^6 M^{-1}
2.4	325.48 (0.13)	-86.66 (0.92)	-0.163 (0.003)	-38.21 (0.40)	4.17 (0.04)
2.8	327.78 (0.16)	-86.66 (0.92)	-0.163 (0.003)	-38.21 (0.40)	4.17 (0.04)
3.0	328.36 (0.15)	-86.66 (0.92)	-0.163 (0.003)	-38.21 (0.40)	4.17 (0.04)
3.5	330.71 (0.17)	-86.66 (0.92)	-0.163 (0.003)	-38.21 (0.40)	4.17 (0.04)

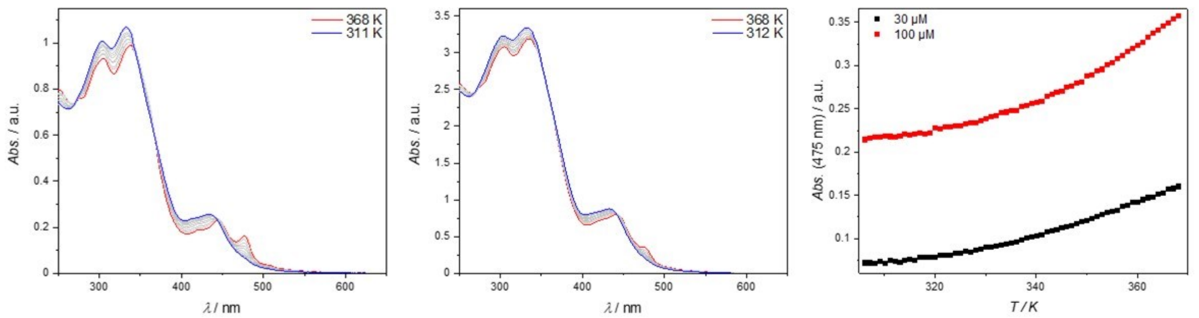

Figure S13: VT-UV/Vis spectra of **1** at *c* =3 x 10^{-5} M (left) and *c* = 10 x 10^{-5} M (middle). Temperature-dependent absorbance at 475 nm (right).

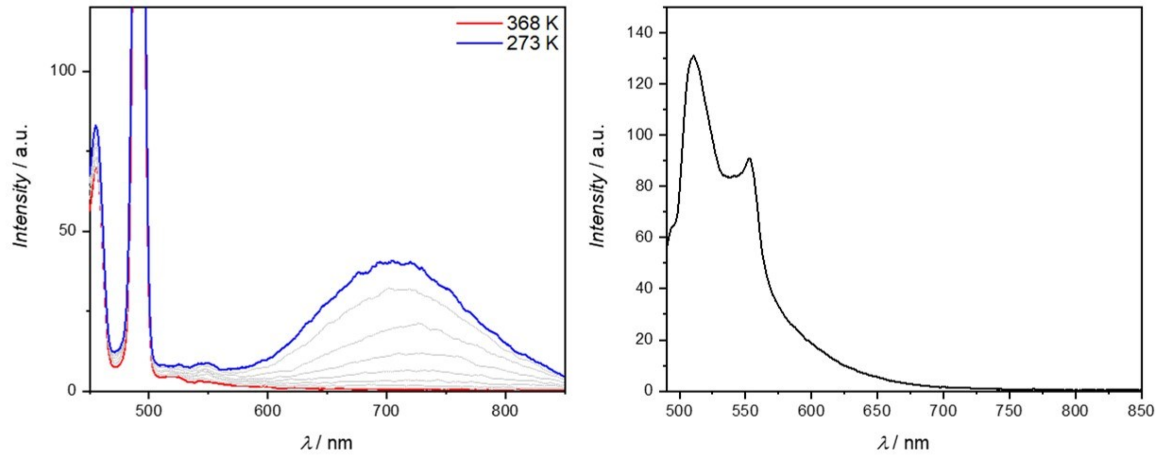

Figure S14: VT-Photoluminescence spectra of **1** (c = 3 x 10^{-6} M, $\lambda_{Exc.}$ = 430 nm) between 368 and 273 K in MCH and emission spectrum of **1** (c = 1 x 10^{-5} M, $\lambda_{Exc.}$ = 475 nm) in chloroform at 298 K. The bands at 460 and 490 nm (left) as well as the peak around 510 nm (right) correspond to artifacts stemming from the solvent (Raman peaks).

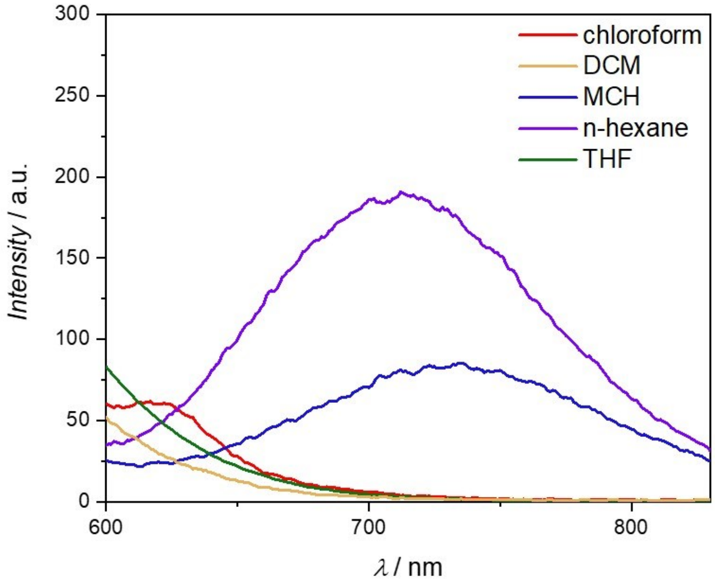

Figure S15: Solvent-dependent photoluminescence spectra of **1** (c = 1 x 10^{-5} M, $\lambda_{Exc.}$ = 420 nm), revealing an emission turn on in the low energy region in aggregation-inducing solvents.

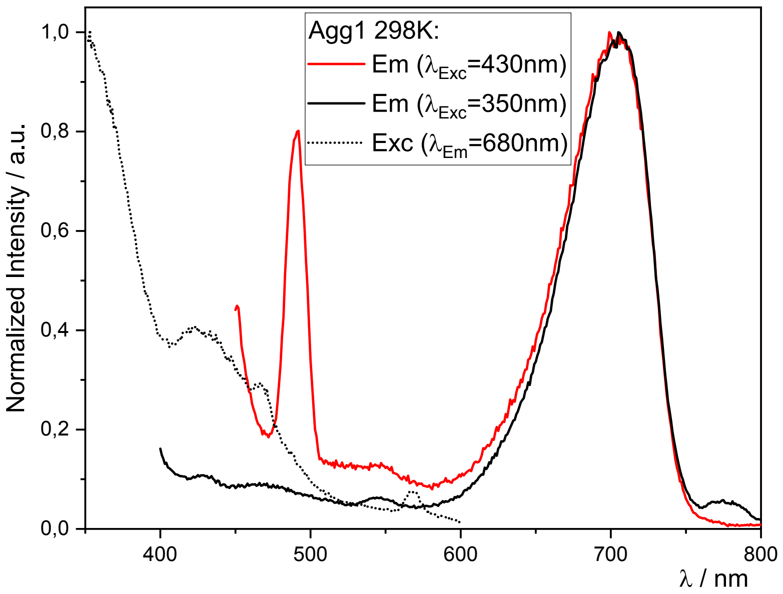

Figure S16: Excitation (dotted line) and emission spectra (solid line) of **Agg1** (λ_{exc} = 350 nm (black); λ_{exc} = 430 nm (red); λ_{em} = 680 nm) at 298 K in MCH. Normalized to the highest intensity. Note that the unusual peak form (steep decline above 720 nm is caused by limitations from the detector, the band at λ_{em} = 490 nm is a Raman peak).

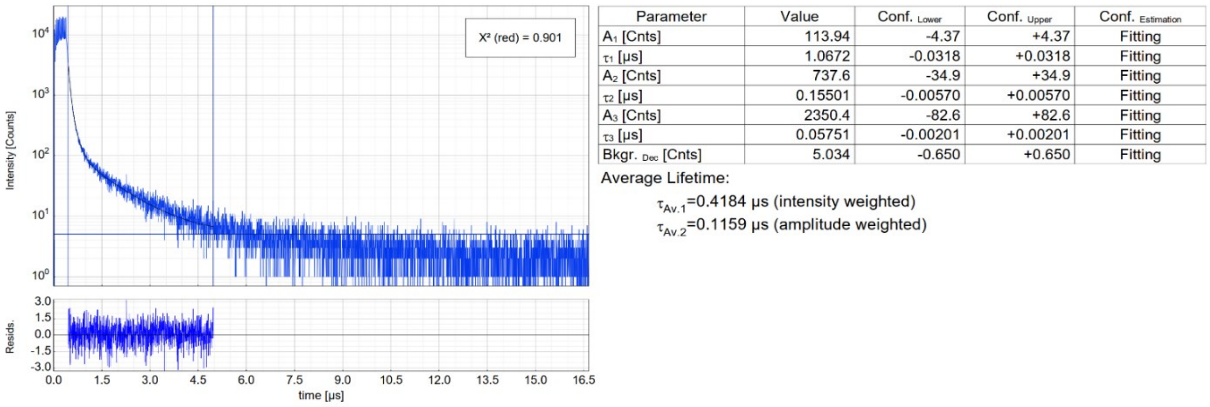

Parameter	Value	Conf. Lower	Conf. Upper	Conf. Estimation
A_1 [Cnts]	113.94	-4.37	+4.37	Fitting
τ_1 [µs]	1.0672	-0.0318	+0.0318	Fitting
A_2 [Cnts]	737.6	-34.9	+34.9	Fitting
τ_2 [µs]	0.15501	-0.00570	+0.00570	Fitting
A_3 [Cnts]	2350.4	-82.6	+82.6	Fitting
τ_3 [µs]	0.05751	-0.00201	+0.00201	Fitting
Bkgr. Dec [Cnts]	5.034	-0.650	+0.650	Fitting

Average Lifetime:

$\tau_{Av.1}$=0.4184 µs (intensity weighted)

$\tau_{Av.2}$=0.1159 µs (amplitude weighted)

Figure S17: Left: Time-resolved photoluminescence decay of **Agg1** in deaerated MCH at 298 K, including the residuals (λ_{exc} = 376.7 nm, λ_{em} = 660 nm). Right: Fitting parameters including pre-exponential factors and confidence limits.

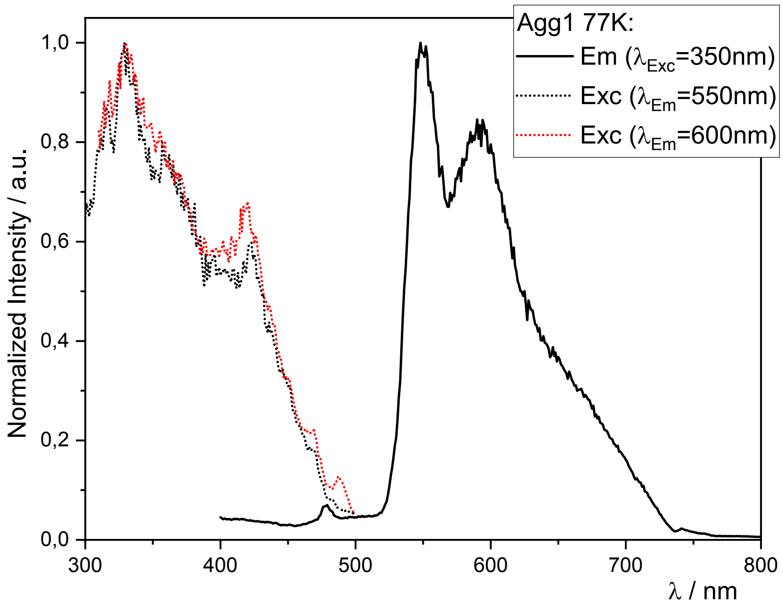

Figure S18: Excitation (dotted line) and emission spectra (solid line) of **Agg1** (λ_{exc} = 350 nm; λ_{em} = 550 nm (black); λ_{em} = 600 nm (red)) at 77 K in MCH. Normalized to the highest intensity.

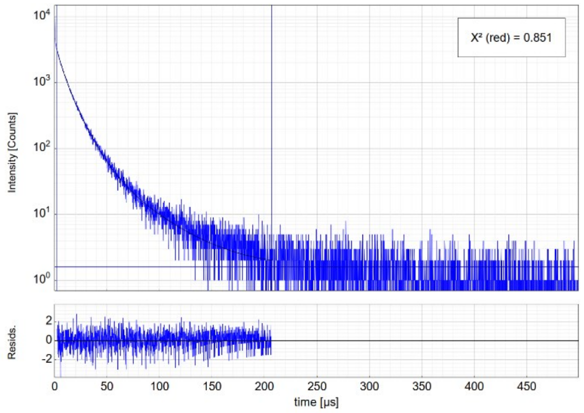

Parameter	Value	Conf. Lower	Conf. Upper	Conf. Estimation
A_1 [Cnts]	120.33	-7.08	+7.08	Fitting
τ_1 [µs]	36.00	-1.18	+1.18	Fitting
A_2 [Cnts]	1704.0	-30.3	+30.3	Fitting
τ_2 [µs]	11.800	-0.160	+0.160	Fitting
A_3 [Cnts]	1265.8	-61.9	+61.9	Fitting
τ_3 [µs]	4.856	-0.251	+0.251	Fitting
Bkgr. Dec [Cnts]	1.604	-0.428	+0.428	Fitting

Average Lifetime:

$\tau_{Av.1}$=13.83 µs (intensity weighted)

$\tau_{Av.2}$=9.90 µs (amplitude weighted)

Figure S19: Left: Time-resolved photoluminescence decay of **Agg1** in glassy MCH at 77 K, including the residuals (λ_{exc} = 376.7 nm, λ_{em} = 550 nm). Right: Fitting parameters including pre-exponential factors and confidence limits.

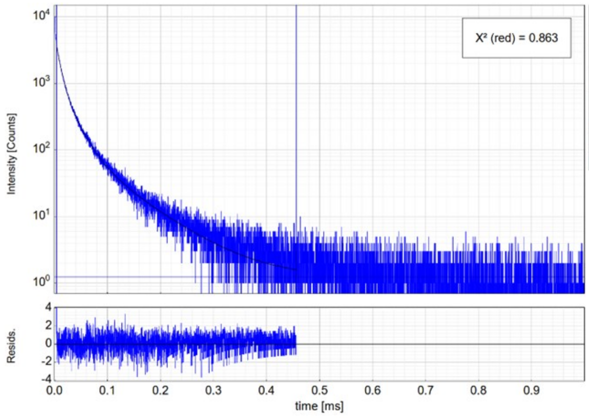

Parameter	Value	Conf. Lower	Conf. Upper	Conf. Estimation
A_1 [Cnts]	137.81	-6.62	+6.62	Fitting
τ_1 [ms]	0.07607	-0.00214	+0.00214	Fitting
A_2 [Cnts]	1074.4	-30.6	+30.6	Fitting
τ_2 [ms]	0.022446	-0.000471	+0.000471	Fitting
A_3 [Cnts]	2545.0	-79.8	+79.8	Fitting
τ_3 [ms]	0.007458	-0.000237	+0.000237	Fitting
Bkgr. Dec [Cnts]	1.2	---	---	<none>

Average Lifetime:

$\tau_{Av.1}$=0.02763 ms (intensity weighted)

$\tau_{Av.2}$=0.01426 ms (amplitude weighted)

Figure S20: Left: Time-resolved photoluminescence decay of **Agg1** in glassy MCH at 77 K, including the residuals (λ_{exc} = 376.7 nm, λ_{em} = 600 nm). Right: Fitting parameters including pre-exponential factors and confidence limits.

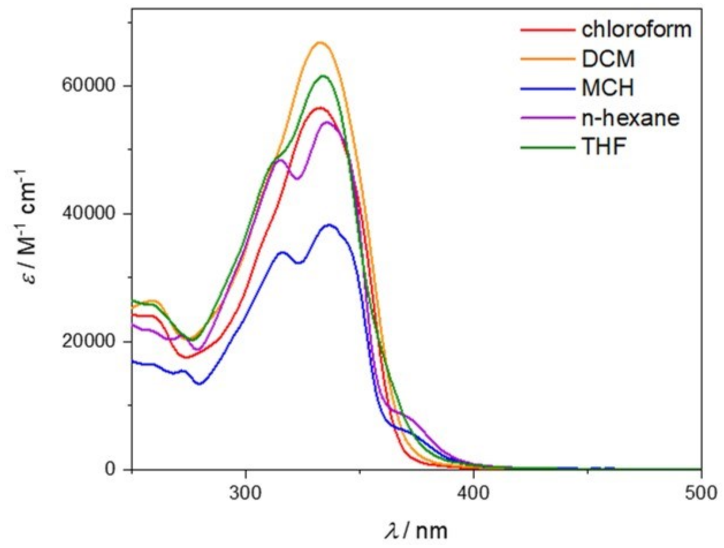

Figure S21: Solvent-dependent UV-Vis absorption spectra of **2** at $c = 1 \times 10^{-5}$ M and 298 K.

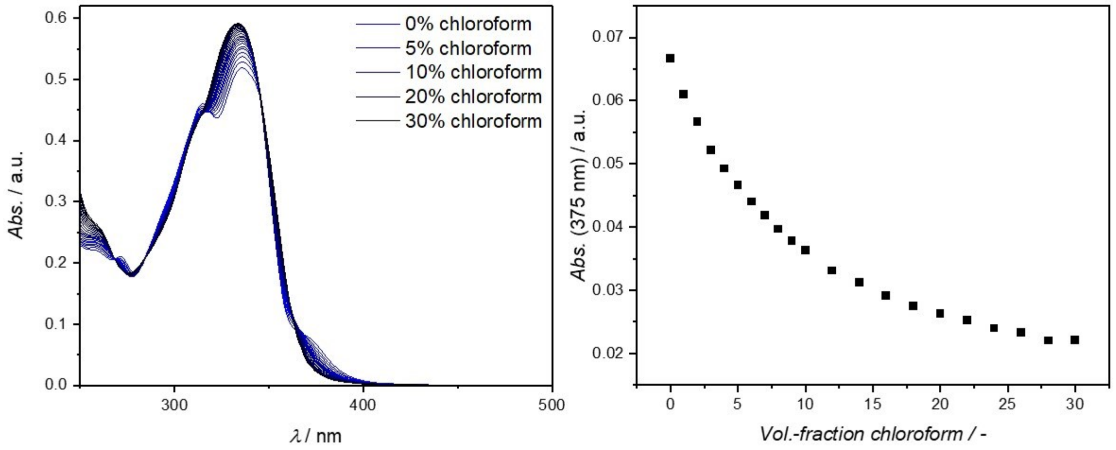

Figure S22: Solvent-dependent UV-Vis absorption spectra of **2** at $c = 1 \times 10^{-5}$ M in different mixtures of MCH and chloroform (left) with the absorbance at λ = 375 nm plotted against the volume fraction of chloroform (right).

Based on the supporting evidence from VT-UV/Vis and solvent dependent NMR, we infer that the observed loss of fine structure upon increase of solvent polarity is caused by an increased stabilization of various rotamers in the OPE backbone as well as the pyridine-Pt bond, which has been previously demonstrated for purely organic OPE based molecules.[8] The band centered around 375 nm in MCH is attributed to the transition into an MLCT state of the molecularly dissolved species in analogy to the assignment for **1**.[9]

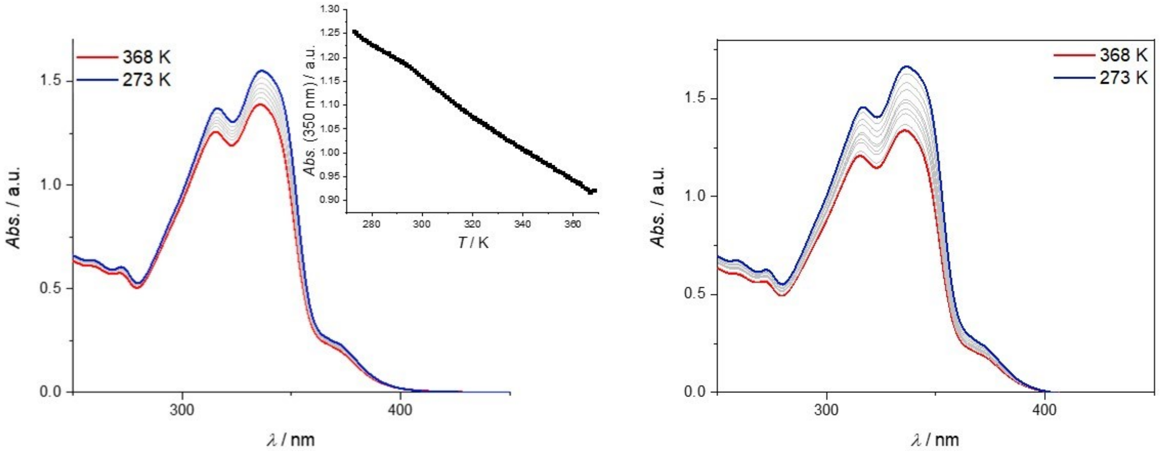

Figure S23: VT-UV-Vis absorption spectra of **2** at c = 3 x 10^{-5} M (left) and 3 x 10^{-4} M (right) between 368 and 273 K in MCH. Inset: Absorbance at λ = 475 nm plotted against the temperature for the measurement conducted at c = 3 x 10^{-5} M.

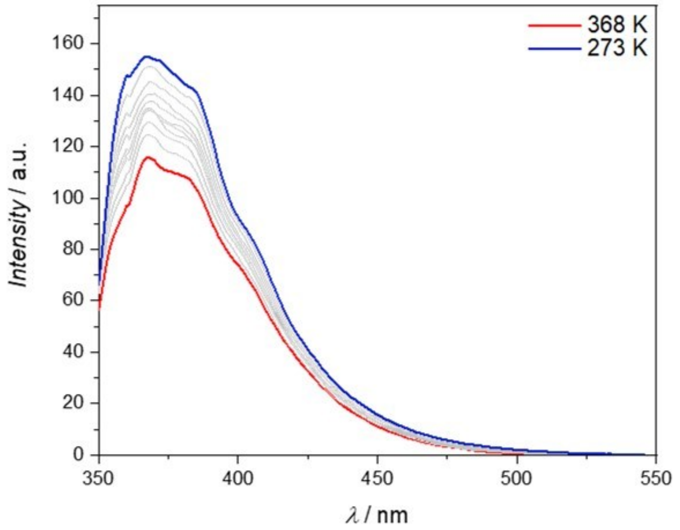

Figure S24: VT-Photoluminescence spectra of **2** (c = 3 x 10^{-5} M, $\lambda_{Exc.}$ = 330 nm) between 368 and 273 K in MCH.

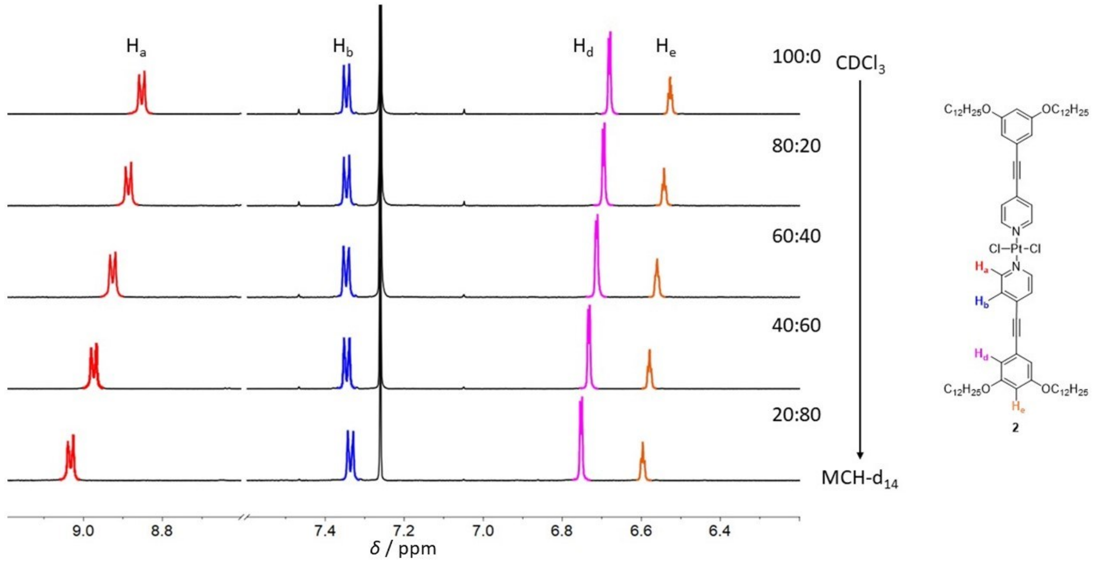

Figure S25: Solvent-dependent ^{1}H-NMR spectra of **2** for different ratios of $CDCl_3$ and MCH-d_{14} at c = 1 x 10^{-3} M and T = 298 K.

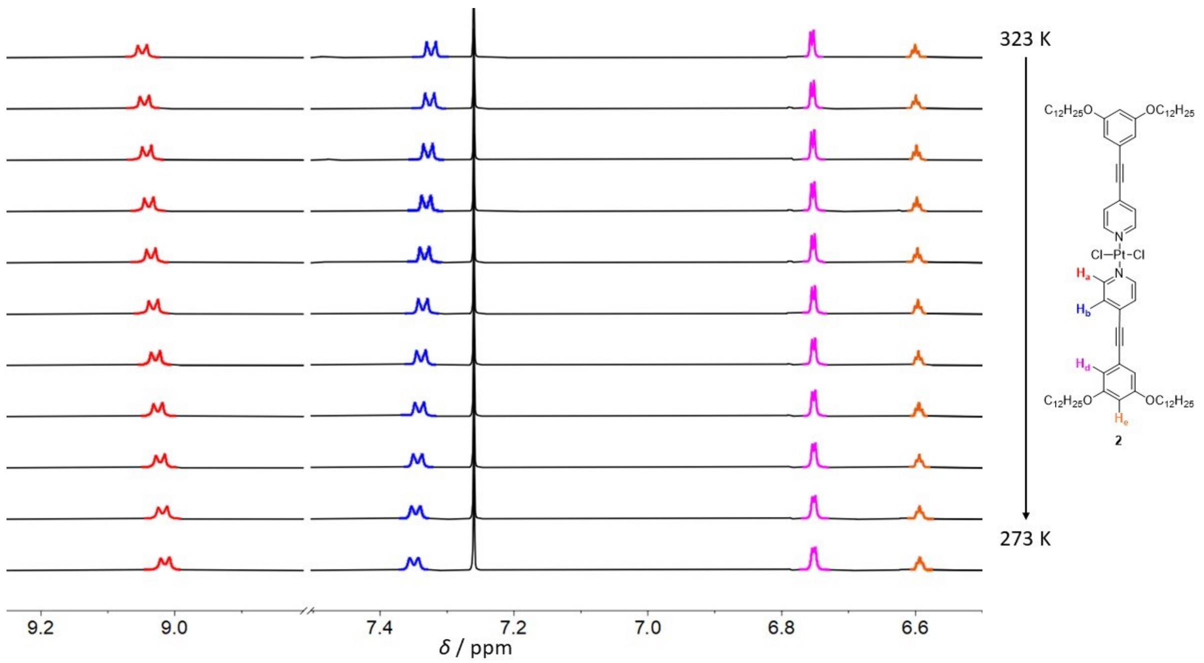

Figure S26: VT-^{1}H-NMR spectra of **2** between 323 K and 273 K in steps of 5 K in MCH-d_{14}/$CDCl_3$ (8:2) at $c = 1 \times 10^{-3}$ M.

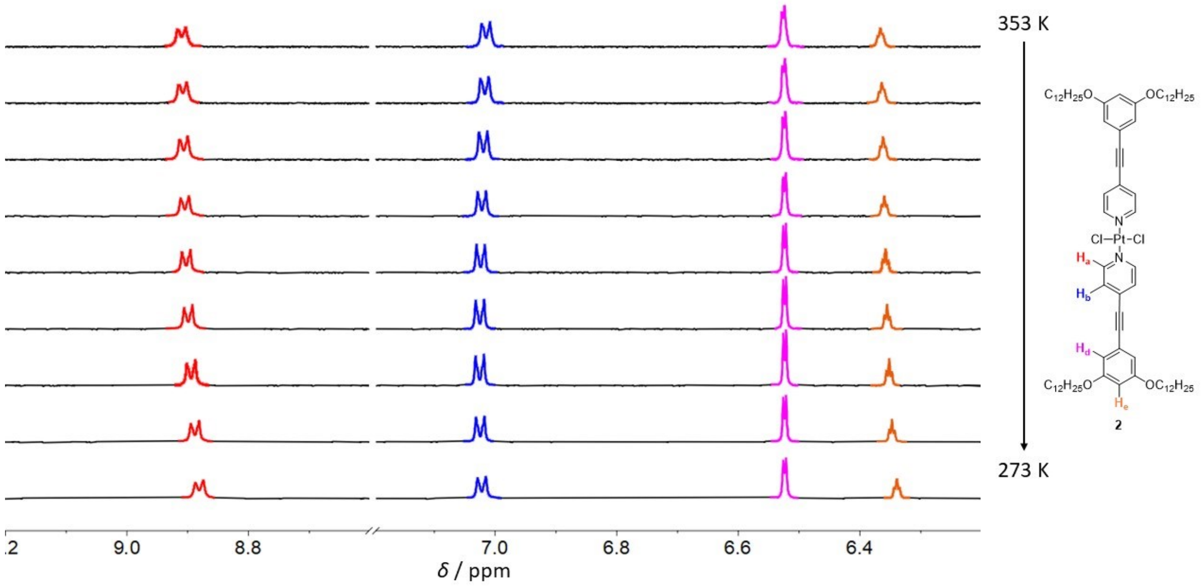

Figure S27: VT-^{1}H-NMR spectra of **2** between 353 K and 273 K in steps of 10 K in MCH-d_{14} at $c = 1 \times 10^{-3}$ M.

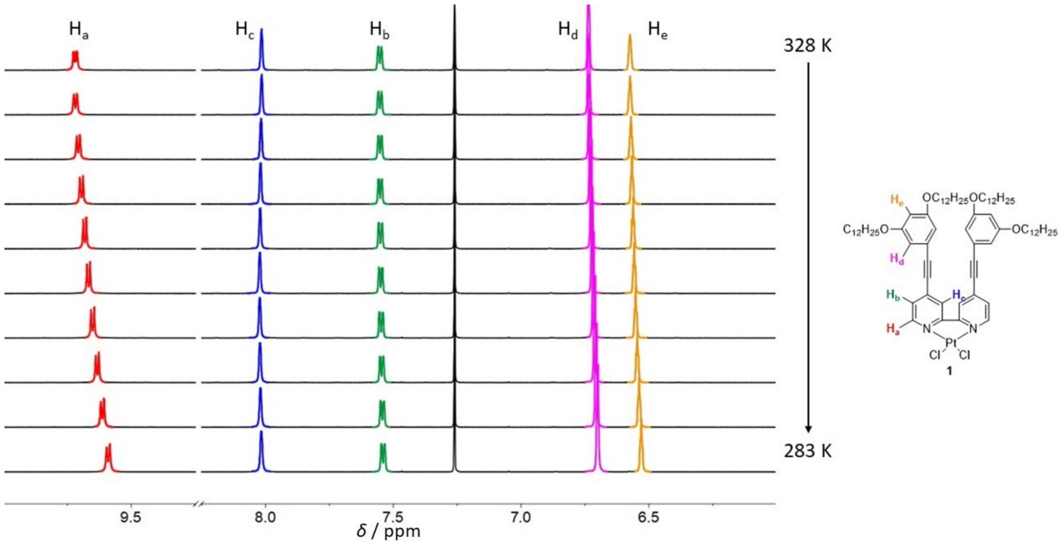

Figure S28: VT-^{1}H-NMR spectra of **1** between 328 K and 283 K in steps of 5 K in $CDCl_3$ at *c* = 1 x 10^{-3} M.

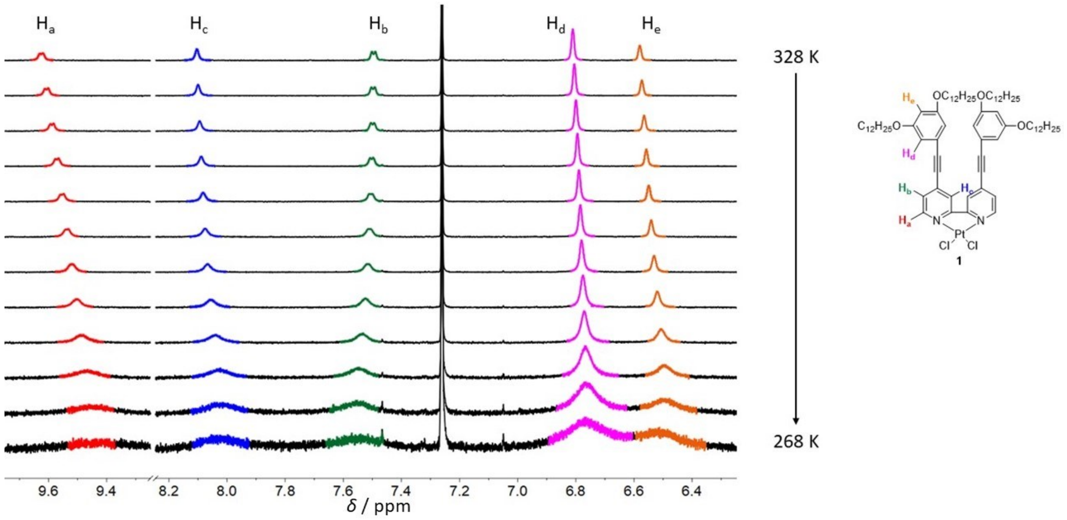

Figure S29: VT-^{1}H-NMR spectra of **1** between 328 K and 268 K in steps of 5 K in $CDCl_3$/MCH (2:8) at *c* = 1 x 10^{-3} M.

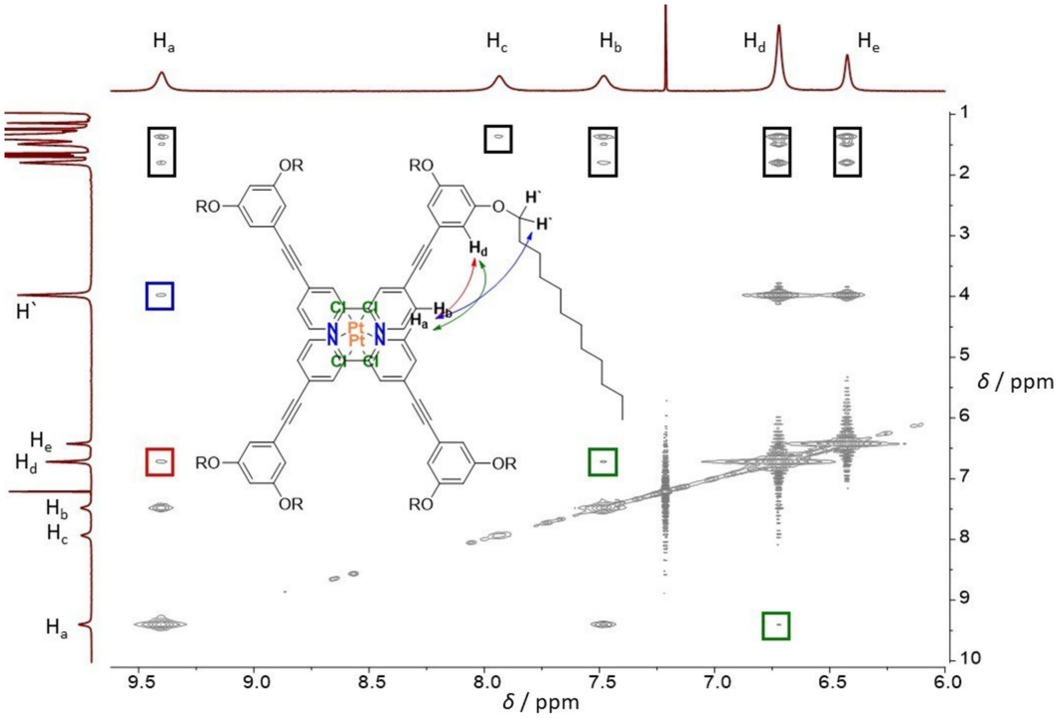

Figure S30: 2D-ROESY-NMR spectrum of **1** in a chloroform/MCH (2:8) mixture at c = 7.5 x 10^{-3} M and T = 298 K. Colored boxes show the most significant intermolecular interactions. Inset: schematic representation of the proposed dimer structure of Agg**1** with the intermolecular close contacts involving aromatic protons indicated with colored arrows.

ROESY NMR studies of Agg**1** reveal numerous intermolecular close contacts. In particular, the alpha proton H_a of the pyridine moiety shows a correlation signal with the proton H_d of the peripheral phenyl ring. In addition, a correlation signal with the first methylene unit of the alkyl chain can also be observed. This interaction is highly unlikely to originate from a parallel 1D stack, or from an intramolecular interaction. Hence, this cross-signal hints at a possible interdigitation of more than one stack in the assembly. Furthermore, the proton H_b gives a correlation signal with the proton H_d as well, although a clear differentiation between inter and intramolecular interactions cannot be made. Various cross-signals between the alkyl chains and the aromatic protons are also appreciable (black boxes). The signals are especially pronounced in the case of the peripheral phenyl ring, possibly through intramolecular close contacts. However, cross-signals with the aromatic protons of the bipyridine moiety can also be observed, indicating an antiparallel arrangement as well as possible interdigitation of the alkyl chains between neighboring stacks. It can also be noted that proton H_c exhibits only relatively weak interactions with the alkyl chains, indicating a shielding effect due to the relative positioning inside of the aromatic backbone.

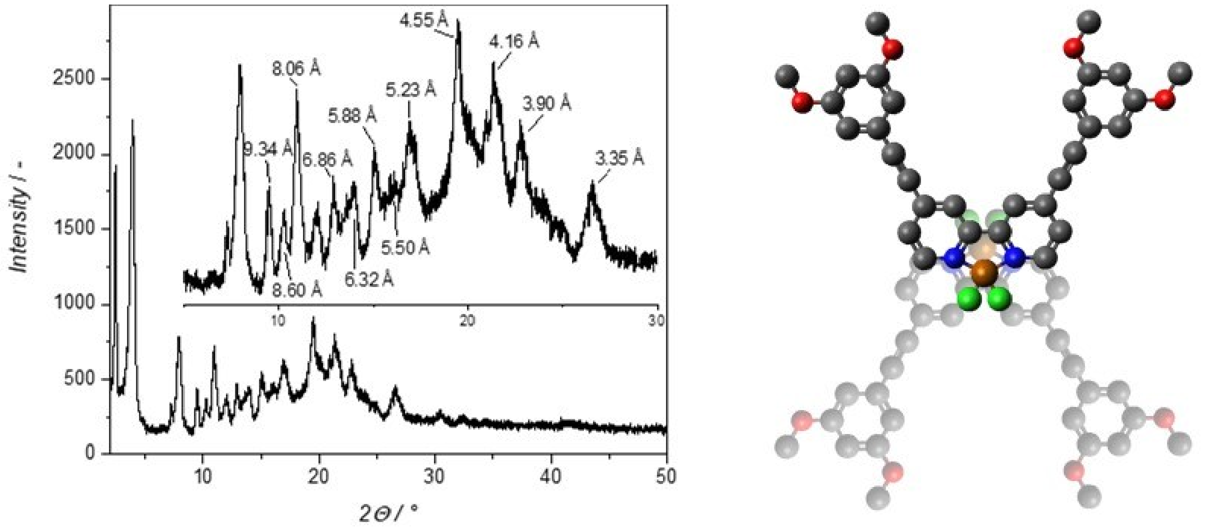

Figure S31: Left: XRD-diffraction pattern (λ = 1.5406 Å) of a thin film of Agg**1** obtained from dropcasting a 7.5 x 10^{-3} M solution (chloroform : MCH 2:8) between 2 and 50° with an inset of the region between 5 and 30°. Right: Schematic representation of the packing mode of Agg**1**. The alkyl chains have been reduced to methyl groups in this representation to enhance visibility.

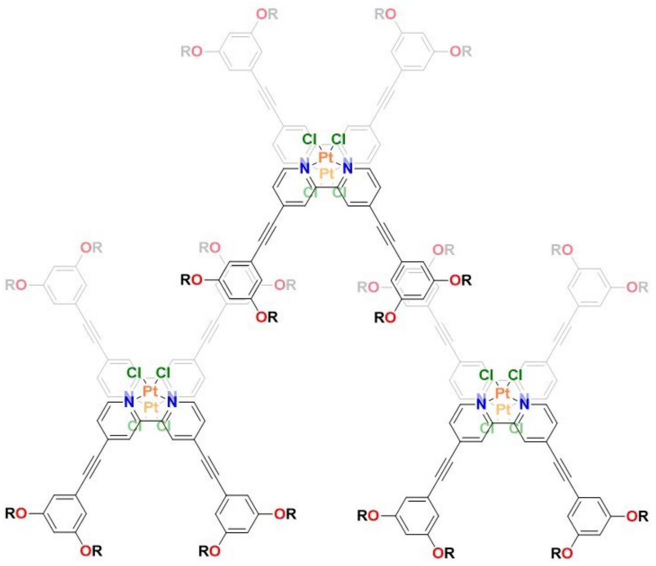

Figure S32: Depiction of the proposed packing mode of Agg**1** in three dimensions (view axis along the antiparallel 1D-stacks). Note that the greyed-out molecules correspond to the layer below the normal colored molecules, while additional layers have been omitted for clarity.

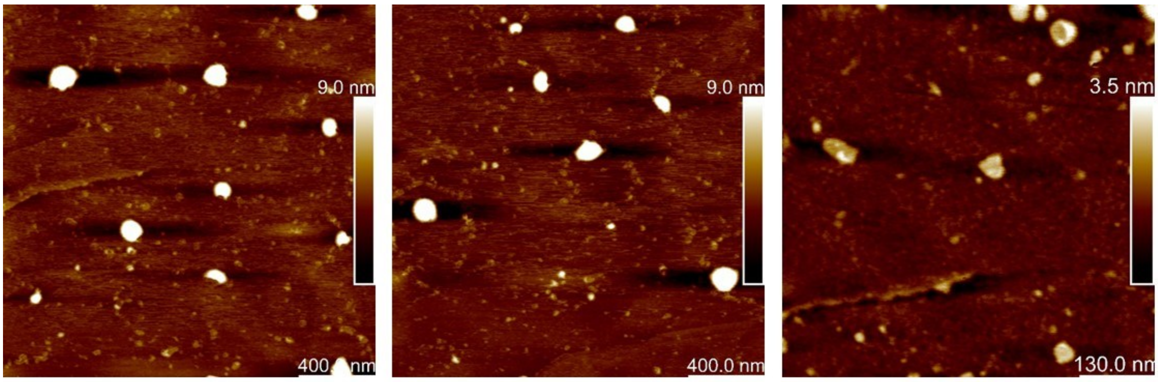

Figure S33: AFM height images of aggregates formed from **1** at c = 5 x 10^{-5} M after cooling from 368 to 273 K in MCH. The samples have been spin coated using a volume of 20 µL.

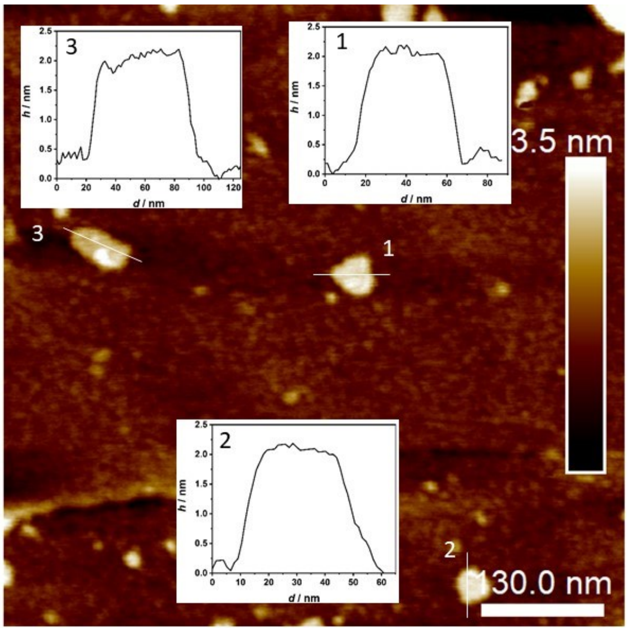

Figure S34: AFM height images of aggregates formed from **1** at c = 5 x 10^{-5} M after cooling from 368 to 273 K in MCH, with the cross-section analysis at various points depicted as insets. The samples have been spin coated using a volume of 20 µL.

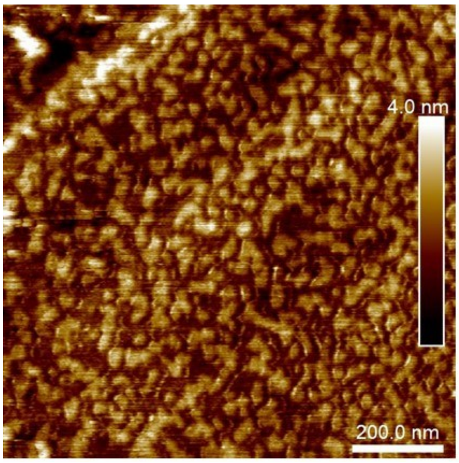

Figure S35: AFM height image of aggregates formed from **1** at *c* = 20 x 10^{-5} M after cooling from 368 to 273 K in MCH. The samples have been spin coated using a volume of 20 µL.

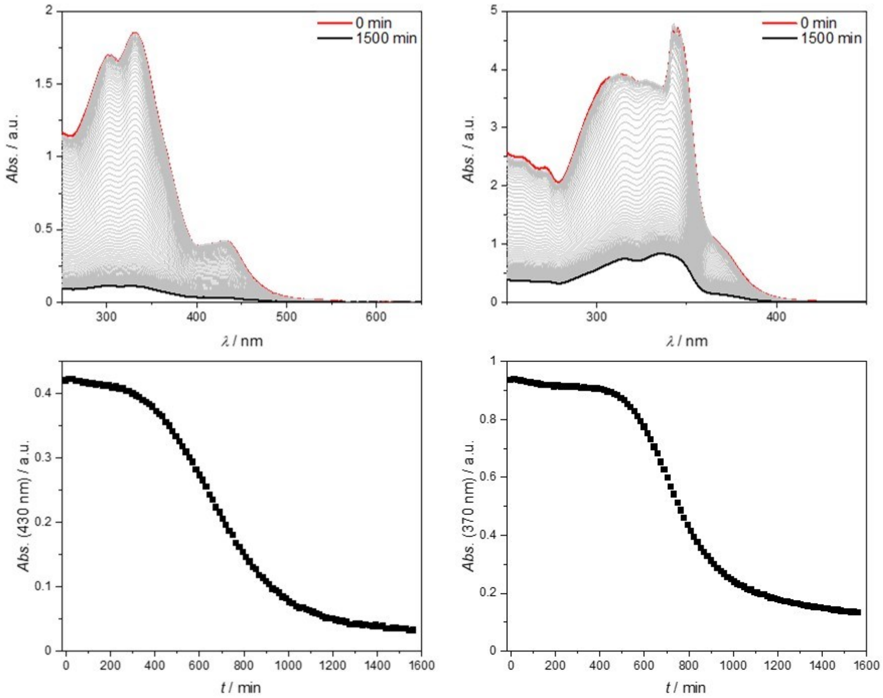

Figure S36: Top: Time-dependent UV/Vis absorption spectra of **1** (*c* = 0.5 x 10^{-3} M, left) and **2** (*c* = 1 x 10^{-3} M, right) at 288 K. Bottom: Time-dependent absorbance of **1** (430 nm, left) and **2** (370 nm, right).

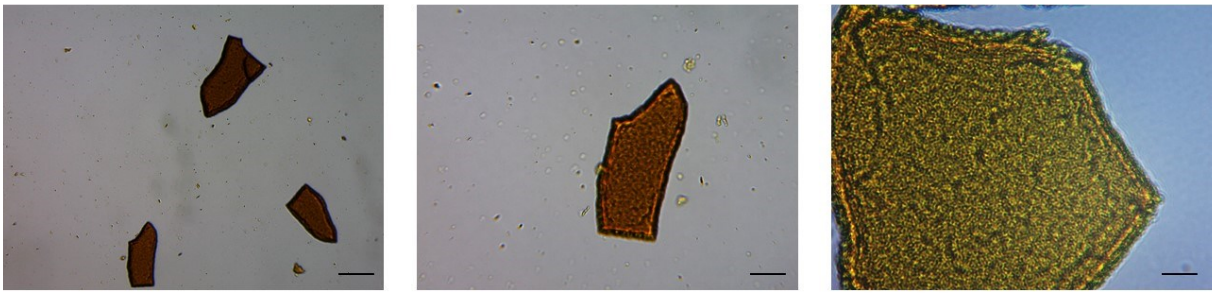

Figure S37: Transmission optical microscopy images of microcrystalline samples of Agg**1**. The scale bars correspond to 250 (left), 80 (middle) and 20 µm (right) respectively.

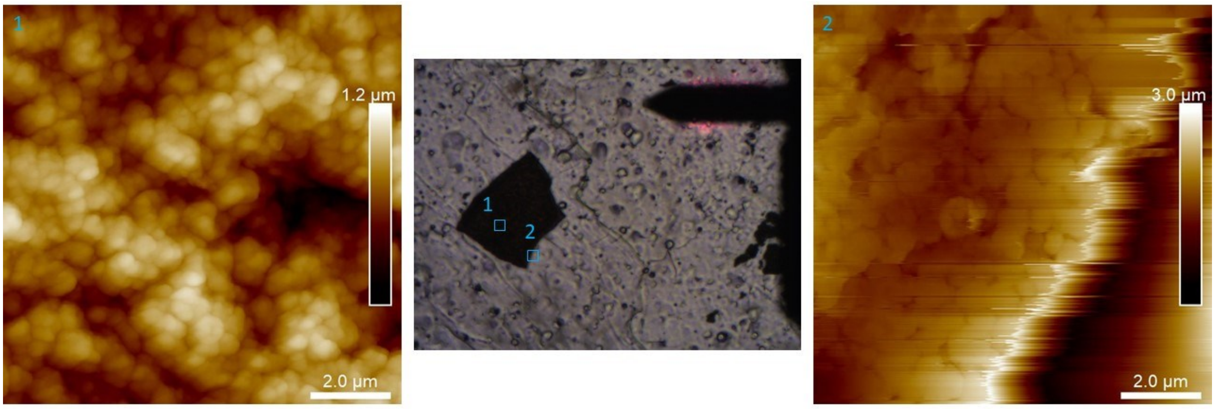

Figure S38: AFM height images of aggregates formed from **1** at *c* = 5 x 10^{-4} M after cooling from 368 to 273 K in MCH. The samples have been spin coated using a volume of 20 µL.

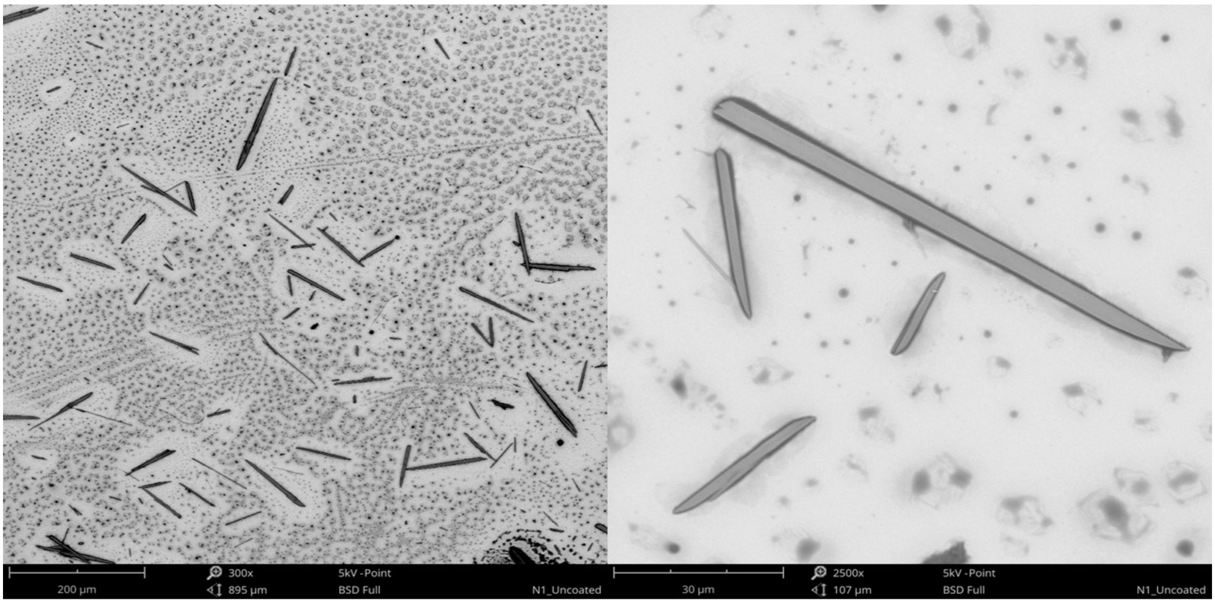

Figure S39: SEM images of Agg**2**. The image sizes are 895 (left) and 107 µm (right) respectively. The samples were obtained after dropcasting roughly 10 µL of solution of **2** in MCH (*c* = 1 x 10^{-3} M) onto a silicon wafer.

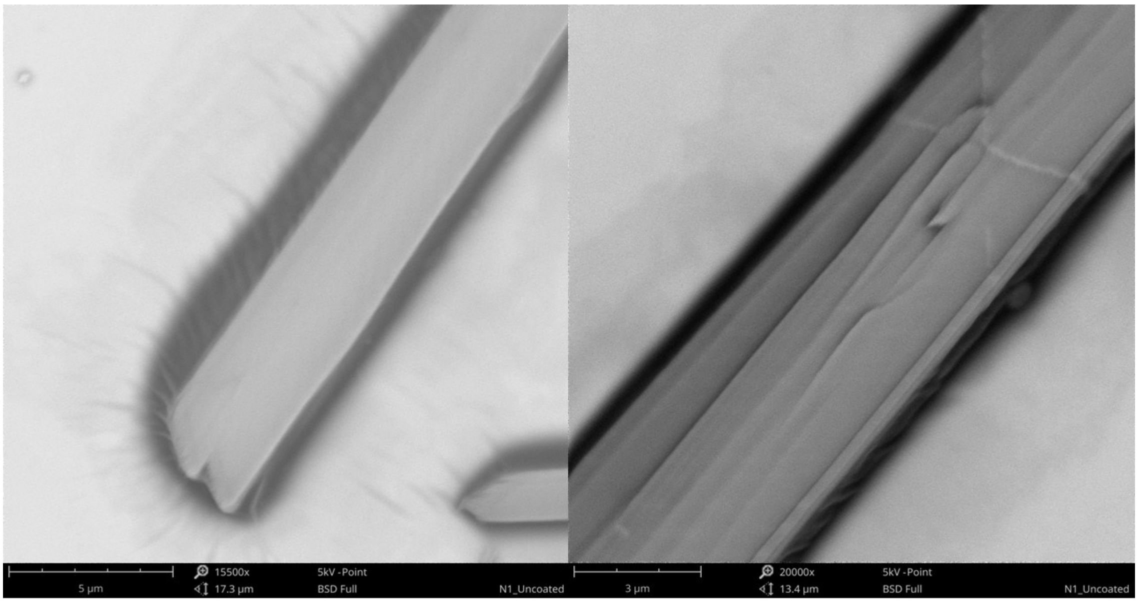

Figure S40: SEM images of Agg**2**. The image sizes are 17.3 (left) and 13.4 µm (right) respectively. The samples were obtained after dropcasting roughly 10 µL of solution of **2** in MCH (*c* = 1 x 10^{-3} M) onto a silicon wafer.

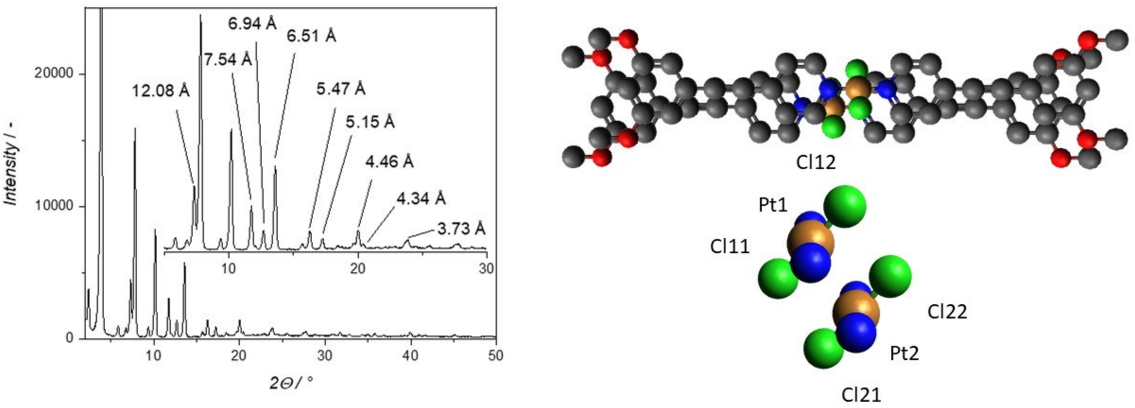

Figure S41: XRD diffraction pattern of Agg**2**. Sample prepared from a solution in MCH (c = 1 × 10^{-3} M) (left) with the proposed molecular packing from a top view (top right) and along the ligands OPE axis (bottom right, ligands reduced to N for clarity).

Comparing this pattern with single crystal X-ray structures of structurally related bispyridyldichlorido Pt(II) complexes,[10] we tentatively assign the reflex corresponding to a distance of 3.73 Å to the intermolecular distance between the aromatic moieties. Additionally, the reflex at 2Θ = 23.86°, correlating to a distance of 4.46 Å, can by assigned to the intermolecular distance between the Pt atoms. Additionally, based on the out-of-plane arrangement of the chlorido ligands (torsional angle = 49°)[10] two separate reflexes corresponding to the intrastrand Pt-Cl distances can be observed (Figure S36). The shorter distance (Pt1-Cl22 / Pt2-Cl11) can be observed at 4.34 Å, while for the second intermolecular Pt-Cl distance (Pt1-Cl21 / Pt2-Cl12) a reflex at 2Θ = 16.29° corresponding to 5.47 Å can be appreciated. In accordance with the single crystal analysis of the structurally related reference compound[10] reflexes corresponding to distances of 6.51, 7.54 and 12.08 Å are attributed to interstrand Pt-Pt distances.

References

(1) N. K. Allampally, A. Florian, M. J. Mayoral, C. Rest, V. Stepanenko, G. Fernández, H-aggregates of oligophenyleneethynylene (OPE)-BODIPY systems in water: guest size-dependent encapsulation mechanism and co-aggregate morphology. *Chem. Eur. J.* 2014, **20**, 10669.

(2) N. Bäumer, K. K. Kartha, S. Buss, I. Maisuls, J. P. Palakkal, C. A. Strassert, G. Fernández, Tuning Energy Landscapes and Metal-Metal Interactions in Supramolecular Polymers regulated by Coordination Geometry. *Chem. Sci.* 2021, **12**, 5236.

(3) L. Wu, M. Eberhart, B. Shan, A. Nayak, M. K. Brennaman, A. J. M. Miller, J. Shao, T. J. Meyer, Stable Molecular Surface Modification of Nanostructured, Mesoporous Metal Oxide Photoanodes by Silane and Click Chemistry. *ACS Appl. Mater. Interfaces* 2019, **11**, 4560.

(4) P. Moreno-García, M. Gulcur, D. Z. Manrique, T. Pope, W. Hong, V. Kaliginedi, C. Huang, A. S. Batsanov, M. R. Bryce, C. Lambert, *et al.* Single-molecule conductance of functionalized oligoynes: length dependence and junction evolution. *J. Am. Chem. Soc.* 2013, **135**, 12228.

(5) a) Y. Sonoda, W. M. Kwok, Z. Petrasek, R. Ostler, P. Matousek, M. Towrie, A. W. Parker, D. Philips, Solvent effects on the photophysical and photochemical properties of (*E,E,E*)-1,6-bis(4-nitrophenyl)hexa-1,3,5-triene. *J. Chem. Soc., Perkin Trans.* 2001, **2**, 308; b) C. Reichardt, Empirical Parameters of Solvent Polarity as Linear Free-Energy Relationships. *Angew. Chem. Int Ed.* 1979, **18**, 98; c) J. P. Cerón-Carrasco, D. Jacquemin, C. Laurence, A. Planchat, C. Reichardt, K. Sraïdi. Solvent polarity scales: determination of new E_T(30) values for 84 organic solvents. *J. Phys. Org. Chem.* 2014, **27**, 512.

(6) P. M. Gidney, R. D. Gillard and B. T. Heaton, Solvent effects on the electronic spectra of some 2,2'-bipyridyl palladium(II) and platinum(II) complexes. *J. Chem. Soc., Dalton Trans.* 1973, 132–134.

(7) M. M. J. Smulders, M. M. L. Nieuwenhuizen, T. F. A. de Greef, P. van der Schoot, A. P. H. J. Schenning, E. W. Meijer, How to distinguish isodesmic from cooperative supramolecular polymerisation. *Chem. Eur. J.* 2010, **16**, 362.

(8) M. Levitus, K. Schmieder, H. Ricks, K. D. Shimizu, U. H. F. Bunz, M. A. Garcia-Garibay Steps To Demarcate the Effects of Chromophore Aggregation and Planarization in Poly(phenyleneethynylene)s. 1. Rotationally Interrupted Conjugation in the Excited States of 1,4-Bis(phenylethynyl)benzene *J. Am. Chem. Soc.* 2001, **123**, 4259.

(9) J. Burgess, Solvent Effects on Visible Absorption Spectra of Bis-(2,2'-bipyridyl)biscyano-iron(II), Bis-(1,10-phenanthroline)bis-cyano-iron(II), and Related Compounds. *Spectrochim. Acta* 1970, **26A**, 1369.

(10) N. K. Allampally, M. J. Mayoral, S. Chansai, M. C. Lagunas, C. Hardacre, V. Stepanenko, R. Q. Albuquerque and G. Fernández, Control over the Self-Assembly Modes of Pt(II) Complexes by Alkyl Chain Variation: From Slipped to Parallel π-Stacks. *Chem. Eur. J.* 2016, **22**, 7810–781.
